# Achieving Ultrahigh DC-Power Triboelectric Nanogenerators by Lightning Rod-Inspired Field Emission Modeling

**DOI:** 10.34133/research.0437

**Published:** 2024-08-13

**Authors:** Qianying Li, Shaoke Fu, Huake Yang, Xiaochuan Li, Xuemei Zhang, Chenguo Hu, Yi Xi

**Affiliations:** Department of Applied Physics, Chongqing Key Laboratory of Materials Physics, College of Physics, Chongqing University, Chongqing 400044, P. R. China.

## Abstract

Direct current triboelectric nanogenerators (DC-TENGs) are a groundbreaking technology to capture micromechanical energy from the natural environment, which is crucial for directly powering sensor networks. However, the research bottleneck in enhancing the triboelectric electrification capability and charge storage capability of dielectrics has hindered the overall performance breakthroughs of the DC-TENG. Here, a field emission model-based DC-TENG (FEM-TENG) is proposed, inspired by lightning rods. The enhanced local electric field between dielectric materials and electrodes induces strong electron tunneling, which improves charge neutralization on the surface of materials and their internal charge storage space, thereby utilizing the dielectric volume effect effectively and strengthening triboelectricity. Guided by the field emission model, the FEM-TENG with a historic crest factor of 1.00375 achieves a groundbreaking record of an average power density of 16.061 W m^−2^ Hz^−1^ (1,591 W m^−3^ Hz^−1^), which is 5.36-fold of the latest DC-TENG. In particular, the FEM-TENG with high durability (100%) truly realizes the collection of breeze energy and continuously drives 50 thermohygrometers. Four additional applications exemplify the FEM-TENG, enabling comprehensive sensing of land, water, and air. This work proposes a paradigm strategy for the in-depth utilization of dielectric films, aiming to enhance the output power of DC-TENGs.

## Introduction

The escalating consumption of fossil fuels and the intensification of global warming pose a threat to the development of human civilization [1]. The advent of ambient energy collectors has greatly mitigated the challenges to human survival posed by the energy crisis [2]. As an energy harvesting technology with strong potential for development, triboelectric nanogenerators (TENGs) demonstrate unique advantages with cost-effectiveness [3], straightforward structures [4], material versatility [5], and flexibility [6], with a wide range of promising applications in micro-nano power supply [7,8], biosensors [9], high-voltage power supply, and blue energy [10–12]. Based on the coupling of triboelectricity and electrostatic induction, the electrification result of TENGs is to produce the pulse alternate current [13–15]. Although the material modification method can effectively improve the performance output of alternate current TENGs, the constant direct current has always been the research focus of energy harvesters [16–20]. Within this realm, the direct current TENG (DC-TENG) stands out as a significant contender, owing to its distinctive advantages such as direct current (DC) output, high integration, resistance to electromagnetic interference, and low crest factor (CF) [21,22]. Furthermore, the CF, which is the ratio of the peak current (or peak voltage) to its root mean square (RMS) value, serves as a crucial metric for assessing output stability [23,24]. In general, the manufacture of the DC-TENG primarily relies on phase control [25], electrostatic breakdown [26], tribovoltaic effect [27], and ternary dielectric triboelectrification effect [28], but the following challenges still remain: (a) Some DC-TENGs, such as those utilizing phase control, still rely on rectifiers for DC output [29,30]. (b) The limited charge accumulation capacity and charge dissipation caused by electrostatic breakdown lead to a notably low average power density in certain DC-TENGs, severely hampering their efficacy in powering electronic devices [31,32]. (c) Built upon triboelectrification, a crucial requirement for achieving high output in DC-TENGs is the intimate contact between triboelectric materials, resulting in severe material wear [26,33]. Therefore, in DC-TENGs, it is difficult to improve energy output and device durability through structural design alone [34]. Moreover, in-depth development of the internal potential of dielectric materials plays a guiding role in improving the overall output of DC-TENGs. In addition, the ability of individual DC-TENGs to harvest ambient energy (wind energy, water energy, etc.) has rarely been explored.

Notably, by utilizing ternary dielectric materials, the DC-TENG exhibits superior advantages in output performance and material selection, with an impressive average power density as high as 6.15 W m^−2^ Hz^−1^ [22,28]. However, the low leakage current and limited charge storage space of dielectric materials limit the performance improvement of the ternary DC-TENG [17]. Recent research indicates not only that polyurethane (PU) foam has ultrahigh leakage charge and robust electronegativity but also that its millimeter-level thickness can effectively prolong the durability of TENGs [35]. The porous network structure in the thick PU foam means that triboelectric charges are no longer completely bound to the surface of the dielectric material, but part of the charges can migrate up and down inside the material, manifesting as the volume effect [35]. Even some TENGs via sliding mode have achieved an impressive output charge density of 5.5 mC m^−2^ by employing the 2-mm-thick PU foam as the electronegative material [36]. However, whether in the alternating current TENG (AC-TENG) or the DC-TENG, the utilization of the PU foam currently remains confined to the dielectric surface effect [35]. Moreover, inefficient utilization of dielectric materials and a superficial understanding of charge transfer between dielectric materials not only impede the enhancement of output and durability in the DC-TENG but also restrict the widespread applications of TENGs. Hence, developing a comprehensive model to deeply explore and utilize dielectric materials and finding the key to restricting the performance breakthrough of DC-TENGs are indispensable conditions for realizing the efficient mechanical energy conversion of TENGs.

Inspired by the lightning rods, for the first time, we propose a field emission model to improve the power density of DC-TENGs, named the FEM-TENG. The model reveals that the local electric field between needle-embedded back electrodes and dielectric films enhances electron tunneling greatly [37–40], thus improving the charge neutralization on the surface of thick dielectric materials and their internal charge storage space. The model not only utilizes the volume effect of the thick dielectric film effectively but also enhances the triboelectric electrification of the entire ternary system. COMSOL simulation, increased leakage current, and the growth in charge density further validate the field emission model. Finally, the FEM-TENG with a CF of 1.00375, obtains an average power density of 16.061 W m^−2^ Hz^−1^ (1,591 W m^−3^ Hz^−1^), setting new records for the DC-TENG reported to date. Moreover, the charge density of the FEM-TENG reaches a historic 9.807 mC m^−2^ (971.6 mC m^−3^). Importantly, leveraging ultrahigh output and exceptional durability (100%), FEM-TENG’s efficacy in harvesting breeze energy is investigated, yielding an unprecedented average power density of 8.461 W m^−2^. In application demonstrations, driven by the breeze (around 2.4 m s^−1^), the FEM-TENG easily lights 3,712 LEDs (no flashing) and continuously powers 50 thermohygrometers. At 120 rpm, the FEM-TENG drives two 50-W lamps, a portable pH meter (rated voltage, 6 V), and a Bluetooth thermohygrometer, respectively. This work establishes a theoretical model for ternary DC-TENGs, utilizing the volume effect of dielectric materials effectively and enhancing its performance comprehensively to harvest environmental mechanical energy.

## Results

### Field emission model based on the electron tunneling effect and working mechanism of the FEM-TENG

Based on the ternary dielectric triboelectrification effect [25], 3 dielectric materials (A, B, and C) with different electronegativity are rubbed in sequence in space, and the electrons generated on material A are transferred to material C through material B, forming the ternary DC-TENG. The specific charge transfer process on the 3 materials is described in Note S1. Our previous work has completed the exploration of the internal mechanism of the ternary DC-TENG basically [22,28]. In this work, we further improve the output performance of the ternary DC-TENG by exploring the inherent potential of dielectric materials. As is well known, to improve stability, we prefer thick dielectric materials as triboelectric materials. However, in ternary DC-TENGs, inefficient utilization of volume effects, limited charge leakage, and unclear models for controlling electrons shuttle between materials pose barriers to achieving performance breakthroughs [22,28,35].

**Fig. 1.**
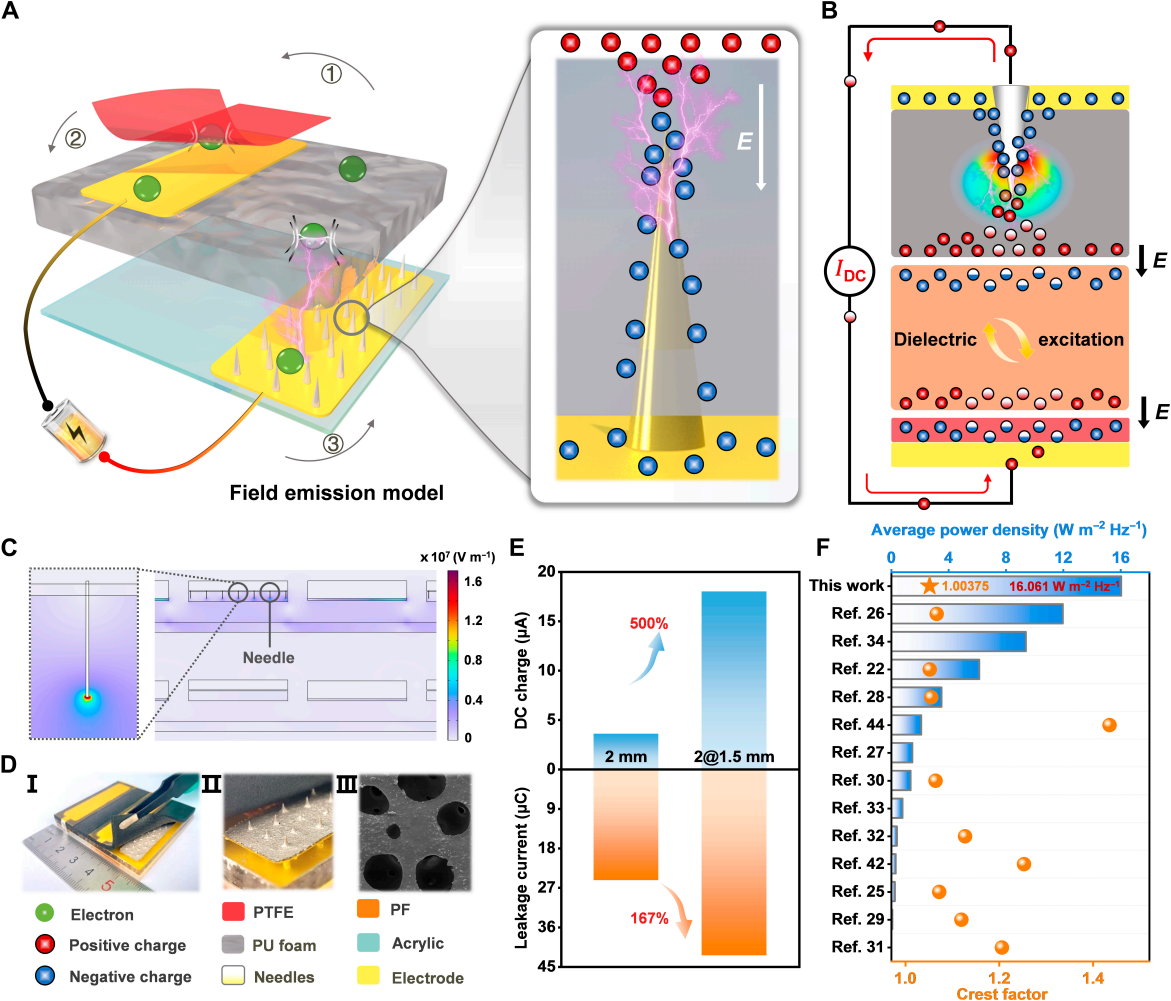
Field emission model, working mechanism, and performance comparison of the FEM-TENG. (A) Concept drawing for the field emission model. It includes the schematic illustration of the 1-unit FEM-TENG. (B) Two-dimensional equivalent model of a ternary system. It explains the charge transfer in the FEM-TENG. (C) Electric field strength between the triboelectric films of the FEM-TENG and the ternary DC-TENG simulated by COMSOL. (D) Physical photograph (inset I) and partially enlarged view of the 2-unit device (inset II). Inset III displays an SEM image of PU foam (scale bar: 100 μm). (E) Comparison of leakage current and DC output charge of the FEM-TENG. (F) Comparison of average power density and crest factor of the FEM-TENG with state-of-the-art works, highlighting the remarkable DC performance of the FEM-TENG.

A lightning rod is a device designed to protect buildings from lightning strikes by harnessing the tip discharge effect (Fig. S1). Its structure provides new insights into designing innovative electrodes. Therefore, we propose a ternary DC-TENG based on a field emission model (FEM-TENG), as depicted in the conceptual diagram of Fig. 1A. The essential components of the field emission model include the high-leakage PU foam (electropositive material), soft polyester fur (PF, intermediate material), smooth polytetrafluoroethylene (PTFE, electronegative material), and the back electrodes embedded with steel needles. Following the triboelectric charging of the 3 materials, the triboelectric charges on PU and PTFE surfaces, along with the induced charges on the back electrodes, create an electric field. According to the potential barrier theory in quantum mechanics [37], the electron tunneling probability *P* is

$$P\propto \text{Eexp}\left[-\frac{\pi^{2}}{\hbar }{\left(2mE_{g}\right)}^{1/2}\left(\frac{E_{g}}{\mathrm{qE}}\right)\right]$$ (1)

where *E* is the electric field intensity between the steel needle electrode and PU interface, *E_g_* is the barrier height, and *ℏ* is the reduced Planck constant. Equation 1 shows that as *E* increases, the probability of electron tunneling experiences a notable increase [37]. Hence, the robust electric field established between the dielectrics and the electrodes induces electron tunneling between them [37,38,40]. Crucially, the synergy of the PU foam featuring a porous structure and the back electrode embedded with steel needles has 3 important effects in the field emission model: Firstly, the steel needles transform electric fields into enhanced local electric fields, thereby initiating the tunneling of a greater number of electrons (right of Fig. 1A) [38,39]. Enhanced electron tunneling triggers extensive charge neutralization on the PU foam surface as well as within it, which manifests as enhanced leakage current. It will be demonstrated in detail in subsequent experiments. Secondly, electrodes with steel needles further expand the charge storage space of the thick PU foam, allowing charges to be stored and induced inside the PU foam (shown as bulk charge), which is an effective strategy to exploit the volume effect of thick dielectric films [35,41]. Thirdly, the enhanced local electric field further stimulates the triboelectric properties of PF and PTFE. In Fig. 1B, the electric field generated by charges on the PU foam excites charges that are different from triboelectric charges on the PF (blue and white negative charges). In the same way, more charges are excited on the PTFE surface, causing more charges to be generated in the external circuit. So far, the field emission model based on the electron tunneling effect has been established, which ultimately enhances the triboelectricity of the ternary system [37,39,40].

In Fig. 1C, the electric field intensity between the triboelectric films of the FEM-TENG (the upper part) and that of a conventional ternary DC-TENG (the lower part) is simulated by COMSOL Multiphysics. More detailed simulation parameter settings and the potential distribution on the triboelectric films are shown in Fig. S2. The pronounced enhanced local electric field (megavolt level) near the tip of the steel needle provides strong theoretical evidence of the field emission [38,39]. Insets I and II in Fig. 1D depict the feature photographs and detailed enlarged view of the 2-unit FEM-TENG. Inset III is a scanning electron microscope (SEM) image of the PU foam, providing evidence of its internal porous structure. The more detailed photographs of the device are presented in Fig. S3. Inserting 1.5-mm steel needle electrodes into the 2-mm PU foam increases the leakage current of the FEM-TENG by 167% and the DC charge output by 500%, validating the field emission model (Fig. 1E).

Before explaining the working mechanism of the FEM-TENG, it should be clear that electrons in the FEM-TENG undergo 3 important shuttles. Initially, leveraging the ternary dielectric triboelectrification effect, electrons are transported from the PU foam to the surface of PTFE through PF (①). Then, due to the tunneling effect, electrons shuttle from the surface of PTFE to the electrode on the back of the PTFE (②). Finally, via the external circuit connection, the electrons tunnel back from the electrode into the PU foam, driven by the enhanced local electric field (③). At this stage, the electrons complete one-way circulation in the FEM-TENG.

Moreover, Fig. S4A depicts a 2-dimensional schematic of the 1-unit FEM-TENG. The PU foam can serve not only as the triboelectric layer of the FEM-TENG but also as the buffer layer, which can enhance the contact efficiency between triboelectric materials. Regardless of whether the slider moves forward or backward, the output current direction of the FEM-TENG remains constant, resulting in a bidirectional DC accumulation phenomenon (Fig. S4B). Before the charges on the triboelectric layer reach saturation, the working mechanism of the FEM-TENG in sliding mode is illustrated in Fig. S5 and further explained in Note S1. Taking the 1-unit sliding structure as an example, Fig. S6 delineates the working mechanism of the FEM-TENG after charges reach saturation. After reaching saturation, in Figs. S5D and S6A, charges on the triboelectric materials induce equal amounts of electrically opposite charges on their corresponding back electrodes through electrostatic induction. Based on the field emission model, owing to the electric field established between triboelectric materials (PTFE and PU foam) and rear electrodes, charge leakage ensues, culminating in charge neutralization (Fig. S6B). Specifically, due to the enhanced local electric field caused by the embedded steel needles in the back electrodes of the PU foam, the leakage current rapidly expands and charge neutralization is enhanced. As the sliding continues, PTFE and the PU foam undergo stronger triboelectrification with the PF, compensating for the neutralized charges (Fig. S6C). As the slider continues its motion, a stronger electrostatic induction occurs on the back electrodes of PTFE and the PU foam, generating a larger DC output in the external circuit (Fig. S6D). Afterward, there is a recurrence of charge leakage enhanced by the local electric field (Fig. S6E). At this juncture, PTFE moves above the PF that has interacted with the PU foam. Owing to the difference in electronegativity between the triboelectric materials [10], electrons on the PF “transfer” to PTFE, mirroring the inherent nature of triboelectricity between the PU foam and PF (Fig. S6F). Hence, a recurrence of electrostatic induction takes place on the stator, yielding a unidirectional DC output (Fig. S6G). As the sliding persists, according to the field emission model, the FEM-TENG undergoes a cycle of charge leakage enhanced by the local electric field, triboelectricity, and electrostatic induction, maintaining the DC output in its external circuit (Fig. S6E to G). While we delineate the working mechanism into stages, it is essential to comprehend that these stages are likely to occur concurrently within a very brief timeframe.

Ultimately, electrons establish a persistent unidirectional cycle throughout the entire device, culminating in a DC output from the FEM-TENG. Theoretical analysis indicates that, owing to the enhanced local electric field, the leakage current between the PU foam and the back electrodes embedded with needles is greatly augmented (Fig. S7). It is beneficial to the subsequent triboelectrification and electrostatic induction of the FEM-TENG. In essence, the enhanced local electric field, induced by steel needles embedded in the electrodes, can increase the number of charges and strengthen the charge migration inside the PU foam, which is a manifestation of effectively utilizing the dielectric volume effect of the millimeter-scale porous PU foam [35], as depicted in Fig. 1E. Remarkably, as is shown in Fig. 1F (the blue bar graph) and Table S1, the optimized FEM-TENG has an average areal power density as high as 16.061 W m^−2^ Hz^−1^ (32.121 W m^−2^). Compared to the state-of-the-art DC-TENG, the FEM-TENG establishes a new record in average power density [22,25–34,42–44]. Moreover, the average areal power density of the FEM-TENG exceeds that of the DC-TENG using the phase control by a factor of 50.19 [42], that of the tribovoltage effect-based DC-TENG by a factor of 10.85 [27], and that of the DC-TENG utilizing the corona discharge by a factor of 1.34 [26]. Even using the PU foam (thickness, 2 mm) as the dielectric material, the average volume power density of the FEM-TENG is as high as 1,591 W m^−3^ Hz^−1^, which is much higher than that of recent DC-TENGs [22,25–34,42–44]. A more detailed performance comparison of the recently published DC-TENG is shown in Table S1. Importantly, in Fig. 1F (the orange symbol graph), the CF of the FEM-TENG achieves the unprecedented value of 1.00375, setting a record for TENG [22,25,26,28–32,42,44].

So far, we have established a field emission model based on the electron tunneling effect, encompassing crucial components, underlying theories, theoretical simulations, and subsequent experimental validations.

### Leakage current characterization and parameter optimization of the sliding FEM-TENG

**Fig. 2.**
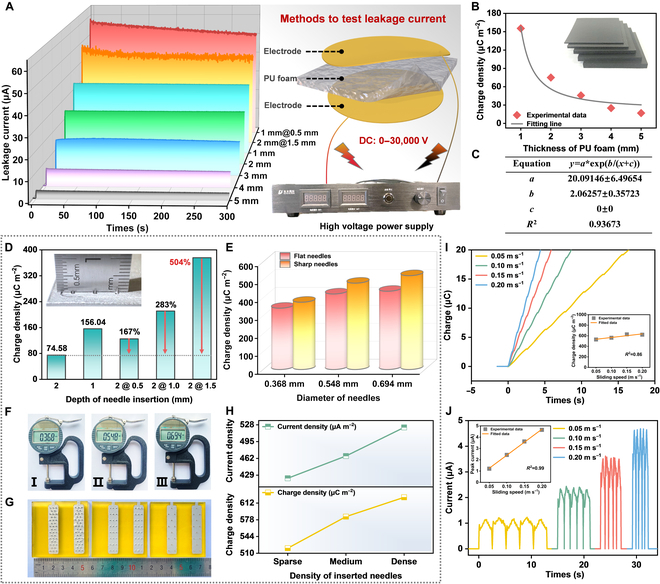
Leakage current characterization and parameter optimization of the sliding FEM-TENG. (A) Leakage current of PU foam with different thicknesses. Among them, the leakage current after adding 0.5-mm and 1.5-mm needles to 1-mm and 2-mm PU foam can also be seen in the figure. The methods and equipment for testing leakage current are in the left part of the figure. (B) Charge density of the 2-unit sliding ternary DC-TENG with different thicknesses of PU foam. (C) The fitting equation for the thickness of PU foam and the charge density of the sliding ternary DC-TENG. (D) Charge density of the sliding FEM-TENG after inserting needles of different lengths into 2-mm PU foam. (E) Effect of needle diameter and needle tip shape on the performance of the sliding FEM-TENG. (F) Photographs of the thickness gauges measuring needles of different diameters. (G) Photograph of electrode substrates with needles of different densities. (H) Performance of the sliding FEM-TENG with different needle densities. (I) Charge and (J) current of the sliding FEM-TENG at different sliding speeds.

To experimentally validate the impact of the electron tunneling effect on the performance output of the conventional ternary DC-TENG, we have measured the leakage current of the PU foam with different thicknesses, as shown in Fig. 2A. As the thickness decreases, the leakage current of the PU foam experiences a notable increase. The PU foam with 1 mm thickness has a leakage current of up to 34.06 μA. The method of testing the leakage current of the PU foam is shown on the right side of Fig. 2A. The high voltage power supply equipment in this work can provide a DC high voltage of 0 to 30,000 V. To emulate the actual conditions of the FEM-TENG, a high voltage of 3,000 V is utilized to test the leakage current of the PU foam. As anticipated, as the PU foam thickness decreases, the leakage current of the PU foam undergoes a sharp increase, resulting in an exponential growth in the charge density of the conventional ternary DC-TENG (Fig. 2B and C and Fig. S8A and B). This is consistent with the results predicted by the relationship between electron tunneling probability and electric field intensity in the process of establishing the field emission model (Eq. 1) [37]. However, acquiring the PU foam with a thickness of less than 1 mm and a high leakage current poses a challenge. Simultaneously, striving to achieve high leakage current characteristics by reducing the thickness of the PU foam will compromise the durability of triboelectric materials, thereby impeding the overall performance improvement of conventional ternary DC-TENGs.

Therefore, the FEM-TENG utilizes the enhanced local electric field to augment the leakage current of the millimeter-level PU foam, effectively utilizing its dielectric volume effect. The electrodes embedded with steel needles are affixed to the back of the PU foam, constituting the sole distinction between FEM-TENGs and conventional ternary DC-TENGs (Fig. S9). Upon embedding the 1.5-mm steel needles in the 2-mm-thick PU foam, a leakage current of 44.51 μA is achieved (Fig. 2A). The greatly increased leakage current proves the enhanced local electric field near the tip and further proves the correctness of the field emission model [38,39]. This PU foam with the specified configuration is referred to as 2@1.5 mm. Furthermore, when the thickness is 1@0.5 mm, the PU foam yields a leakage current of approximately 51.72 μA. Nevertheless, the utilization of the 1-mm-thick PU foam limits the adjustable parameter range; hence, subsequent experiments will employ the PU foam with a thickness of 2 mm. As depicted in Fig. 2D and Fig. S8C, as the length of the inserted steel needle increases, the charge density of the FEM-TENG experiences a sharp increase. The output of the FEM-TENG employing the 2@1.5-mm-thick PU foam is 5 times higher than that of the FEM-TENG with the 2-mm-thick PU foam. Moreover, the charge density of the FEM-TENG rises with the increase in needle diameter, and the sharp needles are more favorable for enhancing charge density (Fig. 2E and F). The enlarged diameter of the steel needles and the sharp tip enhance the probability of electron tunneling [37,38], thereby improving the output of the FEM-TENG. The original charge data plot of the FEM-TENG with needles of different diameters is depicted in Fig. S10A. The schematic diagram of the sharp needle and the flat needle is shown in Fig. S10B. Similarly, elevating the needle density is an effective measure for improving the output of the FEM-TENG. As the density of embedded needles on the electrode increases from sparse to dense, both the charge density and current density of the FEM-TENG exhibit a linear growth pattern (Fig. 2G and H and Fig. S11). In general, factors such as the depth of needle insertion, needle diameter, needle shape, or needle density collectively influence the leakage current of the PU foam by changing the electron tunneling probability in the field emission model, which is ultimately manifested in the output of the FEM-TENG. The manner in which the enhanced local electric field enhances the leakage current of the PU foam is akin to the approach of reducing the thickness of the PU foam, illustrating the effective utilization of the dielectric volume effect.

Hence, the 2@1.5-mm-thick PU foam, the sharp needles with a diameter of 0.694 mm, and the back electrodes embedded with dense steel needles are favorable parameters for the subsequent test of the FEM-TENG. In Fig. 2I, as the sliding speed increases, the optimized FEM-TENG demonstrates minimal variation in its charge output. Therefore, as the lower right inset in Fig. 2I shows, the charge density of the FEM-TENG does not change much either. Surprisingly, the charge density of the optimized FEM-TENG (562.5 μC m^−2^) is increased by 754% compared with the conventional ternary DC-TENG (74.58 μC m^−2^). Additionally, in Fig. 2J, the current of the FEM-TENG shows a linear increase as the sliding speed rises. When the sliding speed is 0.2 m s^−1^, the FEM-TENG can achieve a peak current of 4.68 μA. During this test, the motion parameters of the FEM-TENG are shown in Table S2. Furthermore, as the sliding speed increases, the voltage fluctuation amplitude in the FEM-TENG decreases, maintaining above 3,000 V (Fig. S12A). In Table S3, as the sliding speed increases, the voltage CF of the sliding FEM-TENG is further reduced, which means that the increase in speed can further ensure the constant output of the FEM-TENG. This is consistent with the experimental results reported previously [22,28]. The detailed calculation method of the CF will be shown in the next section. Additionally, according to Fig. S12B and C and Note S2, the average friction force and friction coefficient between the 2-unit FEM-TENG slider and stator are 6.06433 N and 0.3094, respectively, demonstrating relatively smooth movement between the slider and stator.

### Performance characterization of the rotating FEM-TENG

**Fig. 3.**
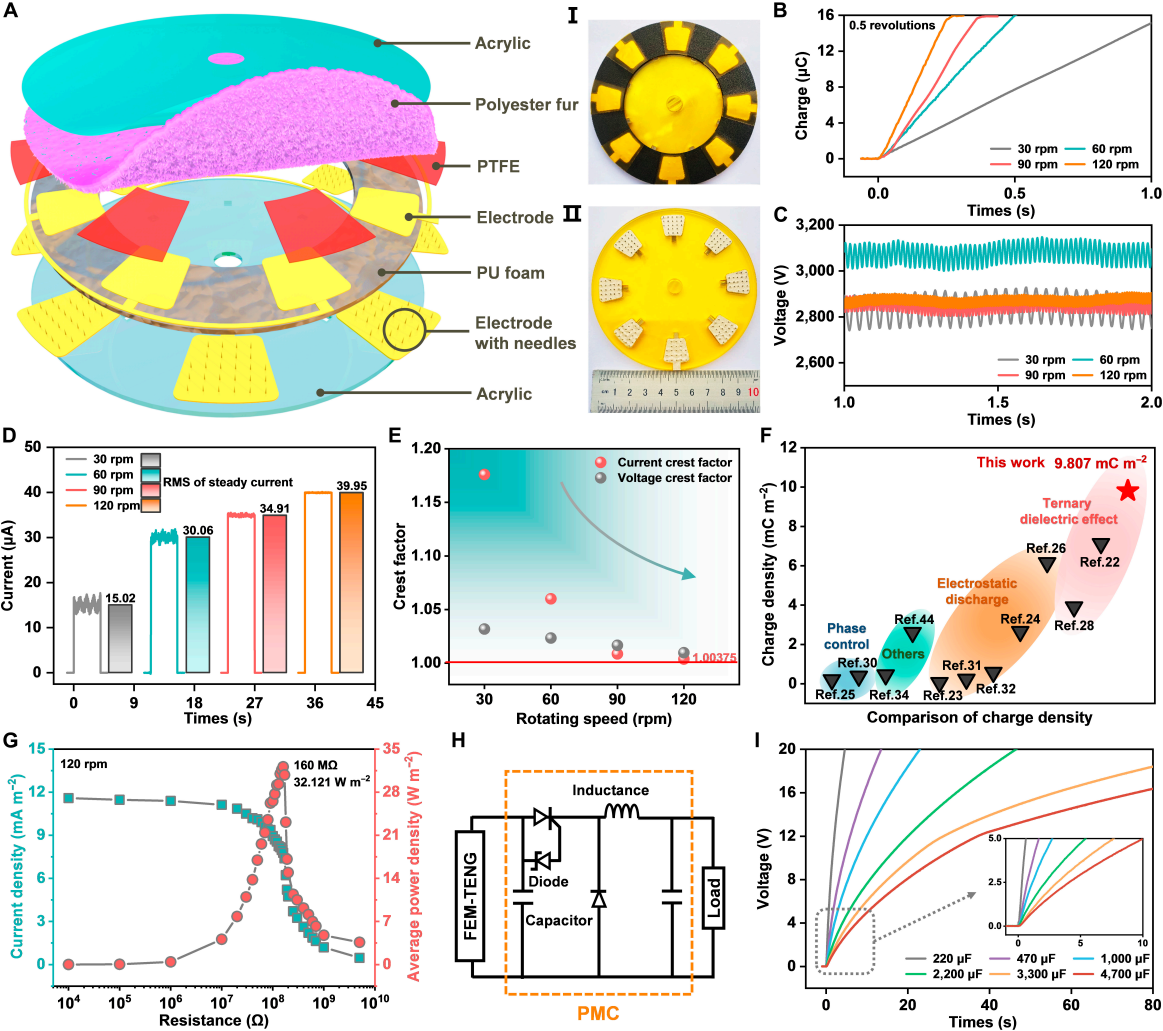
Schematic diagram and performance characterization of the rotating FEM-TENG. (A) Structural diagram of the rotating FEM-TENG. Insets I and II show the stator and the electrode substrate with needles. The diameter of the stator and base plate is 10 cm. (B to D) Output performance of the rotary FEM-TENG at different rotational speeds. (E) Crest factor of the rotary FEM-TENG at different rotational speeds. (F) Comparison of the charge density per round of the FEM-TENG with the latest and most typical DC-TENG. (G) Matching impedance measurements and the maximum average power density output of the FEM-TENG under various external loads. (H) Power management circuit diagram. (I) Voltage curves for fast charging of different capacitors by the FEM-TENG with PMC.

Although prior research has demonstrated that augmenting the number of units can effectively enhance the output performance of the conventional ternary DC-TENG [28], fewer units can better emphasize the impact of the field emission model on the FEM-TENG’s performance. Hence, an 8-unit rotating FEM-TENG is constructed for performance evaluation, and its hierarchical structure is depicted in Fig. 3A. Insets I and II in Fig. 3A show the physical photographs of the stator in the rotating FEM-TENG and the back electrode with embedded needles, respectively. The rotor made of PF is shown in Fig. S13. The rotary FEM-TENG is driven by a stepper motor for standardized testing. With only 0.5 revolutions, the FEM-TENG yields a charge output of up to 16 μC, and its charge output is independent of the rotational speed (Fig. 3B). When the rotation speed is 60 rpm, the DC charge output of the FEM-TENG after rotating 0.5 revolutions is 16.26766 μC, and its raw output data are shown in Fig. S14. As the rotor speed increases sequentially in the FEM-TENG (30 to 120 rpm), the fluctuation amplitude of DC voltage decreases and stabilizes at around 2,800 V (Fig. 3C). According to *I* = *dQ*/*dT* and Fig. 3D, the current of the FEM-TENG rises with the increase in rotation speed. When the rotation speed is 120 rpm, the FEM-TENG achieves an RMS current of 39.95 μA. Furthermore, the equation to calculate the CF of the FEM-TENG is as follows:

$${\mathrm{CF}}_{I}=\frac{I_{\text{peak}}}{I_{\mathrm{RMS}}}$$ (2)

$$I_{\mathrm{RMS}}=\sqrt{\frac{1}{n}\sum_{i=1}^{n}I_{i}^{2}}$$ (3)

$${\mathrm{CF}}_{V}=\frac{V_{\text{peak}}}{V_{\mathrm{RMS}}}$$ (4)

$$V_{\mathrm{RMS}}=\sqrt{\frac{1}{n}\sum_{i=1}^{n}V_{i}^{2}}$$ (5)

where *I_peak_* and *V_peak_* are the peak current and peak voltage, and *I_RMS_* and *V_RMS_* are the RMS current and RMS voltage [23,28]. *CF_I_* represents the CF of the current, while *CF_V_* denotes the CF of the voltage. As rotational speed increases, the peak voltage and peak current of the FEM-TENG gradually stabilize near the RMS voltage and RMS current, leading to a gradual decrease in the CF (Tables S4 and S5). Finally, in Fig. 3E, the FEM-TENG at 120 rpm achieves the lowest current CF of 1.00375, establishing a record for the CF in TENGs (Fig. 1E and Table S1) [22,25,26,28–32,42,44]. The record CF in TENG history proves that the FEM-TENG already has the characteristics of constant current output. Furthermore, according to Fig. S14, the 8-unit FEM-TENG (diameter, 10 cm) can accumulate an impressive DC charge of 32.535 μC per round. As illustrated in Fig. 3F, in comparison to the state-of-the-art rotating DC-TENG [22–26,28,30–32,34,44], the FEM-TENG stands out with an excellent output charge density of 9.807 mC m^−2^ (Table S6). The average charge density of the FEM-TENG is 1.59 times greater than that of the latest published corona discharge-based DC-TENG [26]. Moreover, the volume charge density of the FEM-TENG is 971.6 mC m^−3^, and the comparison of volume charge density in related reported works is shown in Table S6. Figure 3G documents the current density (blue dot line graph) and average areal power density (pink dot line graph) of the FEM-TENG at 120 rpm under varying external load resistances. Moreover, the FEM-TENG has achieved a record-breaking average power density among DC-TENGs [22,25–34,42–44], reaching a maximum value of 32.121 W m^−2^ (average volume power density, 1,591 W m^−3^ Hz^−1^). At this time, the external impedance of the FEM-TENG is 160 MΩ. In Fig. 1E, we have compared the CF and average areal power density of recently published DC-TENGs. Table S1 provides a detailed summary of the relevant important parameters of these DC-TENGs, including average volume power density, average area power density, CF, mechanism and materials, etc. Although the tip structure and thick dielectric film increase the volume of the FEM-TENG, the average volume power density of the FEM-TENG is still higher than that of other DC-TENGs. For example, the average volume power density (1,591 W m^−3^ Hz^−1^) of the FEM-TENG is 1.87 times that of the latest DC-TENG utilizing the charge migration strategy (850.68 W m^−3^ Hz^−1^) [34]. The energy conversion efficiency of the FEM-TENG is 14.27%. The detailed calculation process and compassion of energy conversion efficiency are shown in Note S3 and Table S7. Furthermore, employing a standard power management circuit (PMC) without a rectifier (Fig. 3H), the FEM-TENG effortlessly charges commercial capacitors with different specifications to 5 V within 10 s (Fig. 3I).

Based on the aforementioned outcomes, the significant accomplishments of the FEM-TENG in terms of CF, charge surface density, average areal power density, and average volume power density clearly demonstrate the positive impact of the established field emission model on performance.

### The stability and breeze energy harvesting of the FEM-TENG

**Fig. 4.**
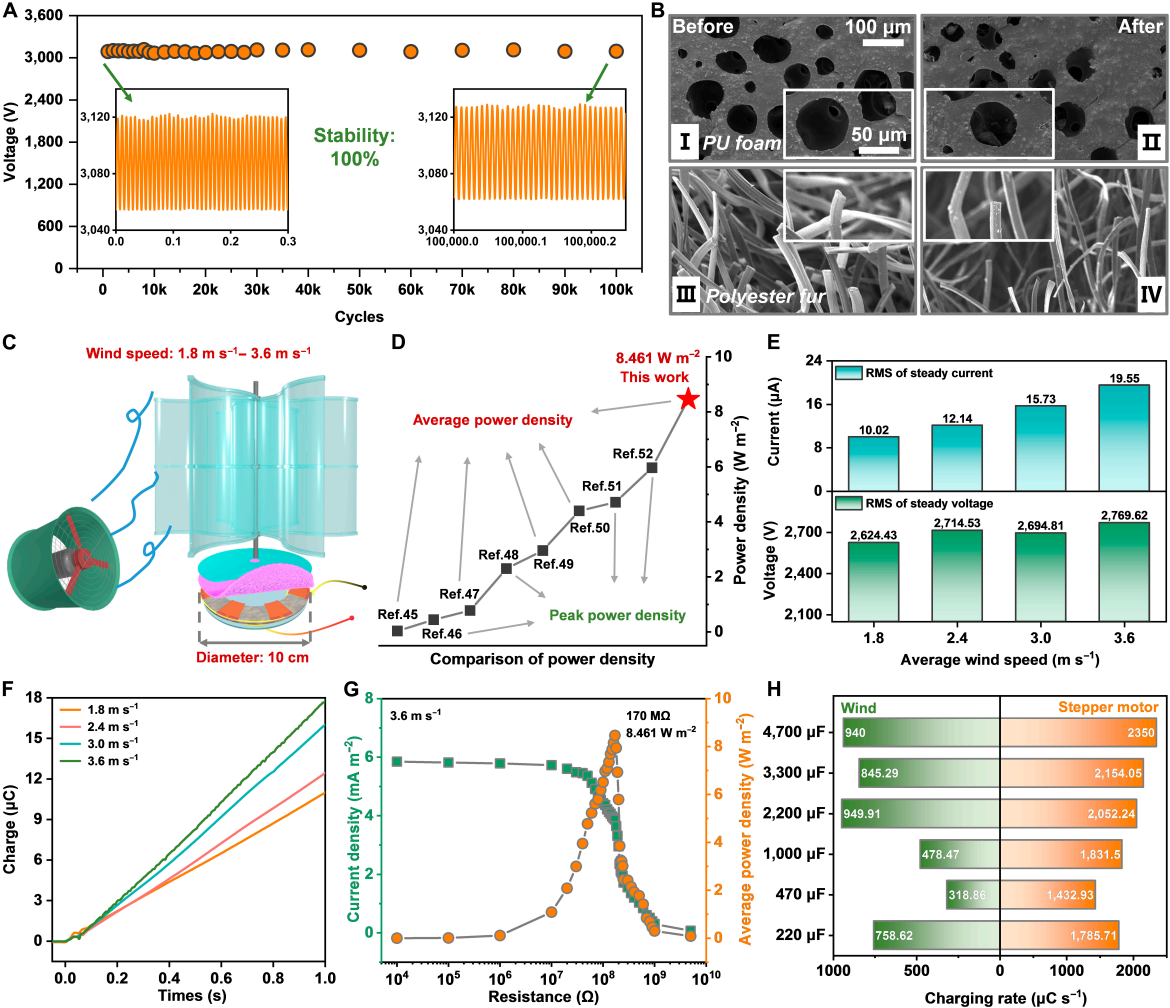
Durability and output performance of FEM-TENG harvesting wind energy. (A) Durability test of the rotating FEM-TENG operating for 100k cycles. (B) SEM characterization of triboelectric materials (PU foam and PF) before and after durability testing, indicating the ultrahigh durability of the FEM-TENG. (C) Schematic illustration of how the FEM-TENG collects wind energy in the laboratory. (D) Comparison of power density between the FEM-TENG and the recent rotary TENG that harvest wind energy. (E and F) Output performance of the FEM-TENG under different wind speeds. (G) Matching impedance and the maximum average power density of the FEM-TENG at a wind speed of 3.6 m s^−1^. (H) Charging rates of various capacitors when the FEM-TENG is driven by wind and stepper motors, respectively.

In general, outstanding output and high durability are essential for broadening the application range of the TENG. The durability test of the FEM-TENG is shown in Fig. 4A. The output of the rotating FEM-TENG remains nearly constant over 100k operating cycles, reaching 100% stability. The exceptional durability of FEM-TENGs can be attributed to the smooth texture of PTFE, the millimeter-level thickness and perfect elasticity of the PU foam, and the soft tip of PF. Table S8 records the changes in the voltage CF of the FEM-TENG before and after the stability test. Long-term operation reduces the CF to 1.0109, which is also the result of the soft PF polishing the PTFE and the PU foam [28]. Figure S15A displays the raw data from the stability test. Simultaneously, before and after the durability test, SEM images of the 3 triboelectric materials reveal no significant surface wear, reaffirming the exceptional robustness of the FEM-TENG (Fig. 4B and Fig. S15B).

To showcase commercial viability, in Fig. 4C, a wind energy collection system is assembled using a homemade wind cup, a blower, and the rotating FEM-TENG. As shown in Table S9, the blower’s wind speed is adjustable via input voltage, with an average range spanning from 1.8 to 3.6 m s^−1^. The RMS current of the FEM-TENG increases linearly as the wind speed rises (Fig. 4E and Fig. S16A). When the wind speed is 3.6 m s^−1^, the RMS current attains 19.55 μA. The RMS voltage of the FEM-TENG remains relatively stable across varying wind speeds, with a value of around 2,700 V (Fig. 4E and Fig. S16B). The CFs of the FEM-TENG during the wind energy harvesting process are documented in Table S10 (current CF) and Table S11 (voltage CF). Even during wind driving, the operation of the FEM-TENG is not as smooth as motor driving, but all CFs are below 1.07. Moreover, in Fig. 4F, as wind speed increases, the rate of DC charge accumulation in the FEM-TENG within 1 s also rises. When the wind speed is around 3.6 m s^−1^, the FEM-TENG accumulates 18 μC DC charge in 1 s. Subsequently, in Fig. 4G, the FEM-TENG obtains an average power density of 8.461 W m^−2^ with an external resistance of 170 MΩ (wind speed, 3.6 m s^−1^). Upon comparison with the latest research [45–52], it indicates that the average power density of the FEM-TENG outperforms that of other wind energy-driven rotating TENGs, underscoring its exceptional performance as an ambient mechanical energy harvester (Fig. 4D and Table S12). Furthermore, in Fig. 4H and Fig. S17, driven by a wind speed of 3.6 m s^−1^, the FEM-TENG charges the commercial capacitors (220 μF/470 μF/1,000 μF/2,200 μF/3,300 μF/4,700 μF) with PMC (no rectifier). Specifically, the wind-driven FEM-TENG reaches a charging rate of 949.91 μC s^−1^ for a 2,200-μF capacitor, while the motor-driven FEM-TENG achieves an even higher charging rate of 2,350 μC s^−1^ for a 4,700-μF capacitor (Fig. 4H and Table S13). However, in general, the wind-driven FEM-TENG exhibits a lower output performance compared to its motor-driven counterpart. This is attributed to the instability of the rotor in the FEM-TENG when driven by the wind cup, resulting in inadequate contact between the triboelectric layers and consequently reducing the overall output. Crucially, the stability test and performance in the wind energy collection process have convincingly demonstrated the practical value of the FEM-TENG.

### Self-powered all-around sensing system realized by the FEM-TENG and its demonstration

**Fig. 5.**
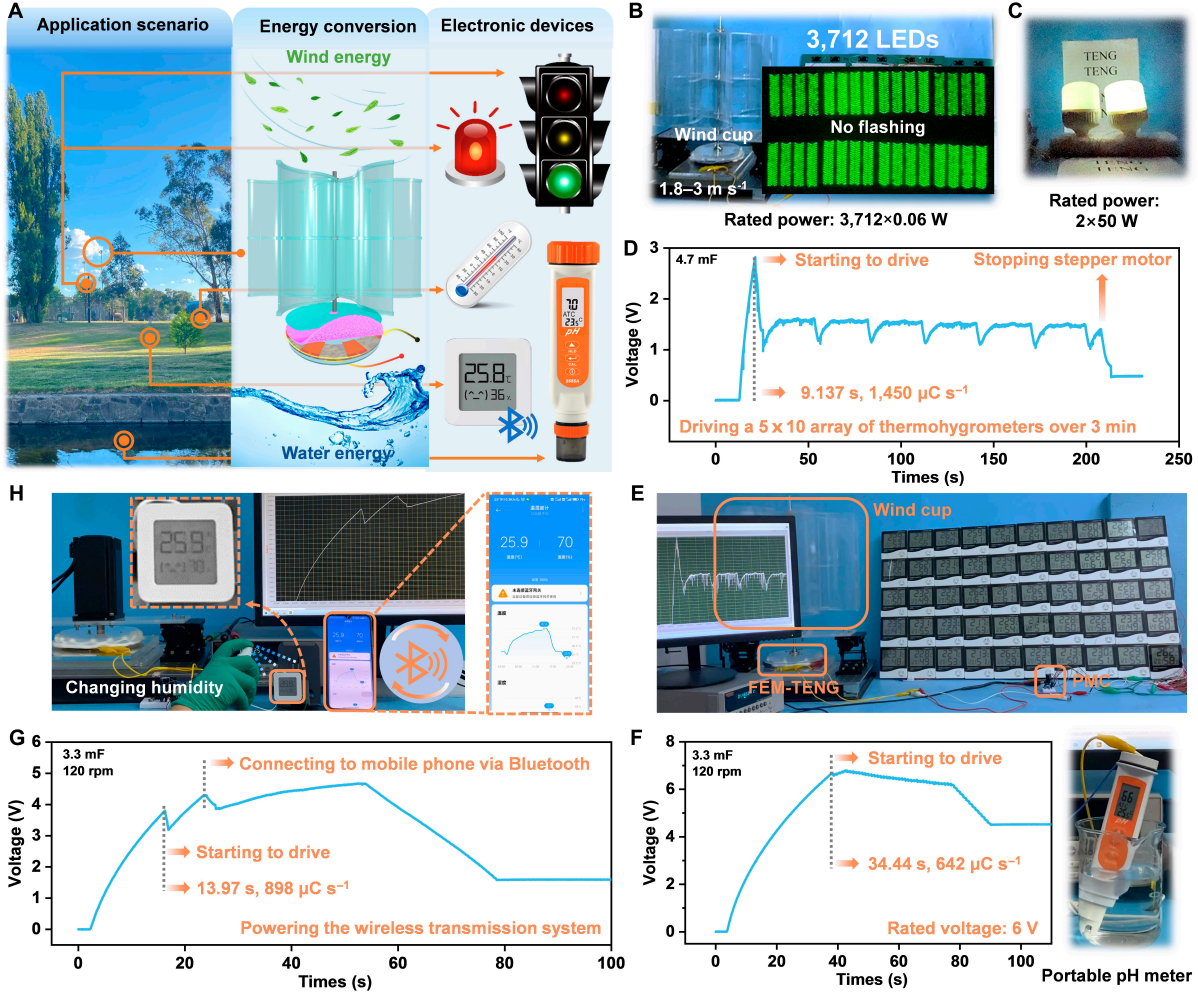
Application demonstration of the FEM-TENG in a self-powered all-around sensing system. (A) Application scenario of the all-around sensing system based on the FEM-TENG driven by ambient mechanical energy. (B) A total of 3,712 LEDs (diameter, 5 mm) lighted directly by driving the FEM-TENG at a wind speed of 1.8 to 3 m s^−1^. (C) Two lamps (50 W each) lighted directly by the wind-driven FEM-TENG. No flashing LEDs and lamps demonstrate the ultralow crest factor of the FEM-TENG without PMC. (D) Voltage–time curve of 50 thermohygrometers powered by the wind-driven FEM-TENG with PMC. (E) Realistic scene of the FEM-TENG powering a sensing array of 50 thermohygrometers by collecting wind energy. (F) Voltage–time curve of a portable pH meter (rated voltage: 6 V) powered by the FEM-TENG at 120 rpm. (G and H) Realistic scene and voltage–time curve of a Bluetooth thermohygrometer powered by the FEM-TENG at 120 rpm. The values of ambient temperature and humidity can be transmitted to the mobile terminal in real time.

Depending on the conducted tests, there is strong evidence supporting the FEM-TENG’s capability to harness wind or water energy from the environment through various triggers, thereby completing the energy conversion process. Building upon the exceptional DC output of the FEM-TENG, we propose a self-powered all-around sensing system that encompasses a majority of electronic sensing devices within a park, as shown in Fig. 5A. Furthermore, fundamental warning lights, temperature and humidity sensing arrays, wireless Bluetooth transmission equipment, water quality detection, and other devices can be effortlessly powered by the FEM-TENG without any rectifier.

Thanks to its constant DC and high voltage output, the 10-cm-diameter FEM-TENG can harness wind at around 2.4 m s^−1^ to illuminate 3,712 LEDs directly (Fig. 5B). Throughout the lighting process, the LEDs with diameters of 5 mm exhibit no flashing, as shown in Movie S1. Additionally, the rotating FEM-TENG directly drives two 50-W lamps, providing continuous illumination for the presentation space (Fig. 5C and Movie S2). Moreover, the FEM-TENG can also be combined with other energy harvesters (such as solar cells) to power high-energy-consuming electronic devices such as traffic lights, preparing for the realization of all-around and all-weather self-powered signal sensing.

In Fig. 5D and Movie S3, the FEM-TENG harnesses breeze energy to power a 5 × 10 array of thermohygrometers for over 3 min, assisted by PMC and a 4,700-μF capacitor (no rectifier). In this process, the FEM-TENG charges the 4,700-μF capacitor to 2.819 V in 9.137 s, achieving a remarkable charging rate of 1,450 μC s^−1^. The application scenario where 50 thermohygrometers are driven is shown in Fig. 5E. Furthermore, the combination with supercapacitors can effectively solve the randomness and discreteness problems of the FEM-TENG in converting wind energy [53]. Moreover, in Fig. 5F and Movie S4, the FEM-TENG with PMC (no rectifier) successfully drives a portable pH meter with a rated voltage of 6 V at 120 rpm, establishing a FEM-TENG-based water quality detection sensor. Furthermore, to showcase FEM-TENG’s capability to power the wireless sensing system, a 10-cm-diameter device with PMC (no rectifier) is employed to drive a Bluetooth-enabled thermohygrometer with a rated voltage of 3 V (Fig. 5G and Movie S5). Hence, the FEM-TENG-based wireless transmission system can provide real-time sharing of abrupt changes in temperature and humidity within the environment on the smartphone terminal (Fig. 5H). Until now, the FEM-TENG has leveraged its ultrahigh DC output to accomplish comprehensive sensing across land, water, and air.

## Conclusion

In summary, we have successfully converted the ambient mechanical energy into constant output by developing a ternary DC-TENG based on the field emission model. First, the field emission model has disclosed the electron tunneling effect between dielectric materials and electrodes. COMSOL simulations and material leakage current tests have further indicated that the local electric field can amplify electron tunneling. Consequently, the back electrodes with steel needles and the millimeter-thick PU foam are employed to optimize the FEM-TENG. This ingenious combination enhances the charge neutralization on the surface of the millimeter-thick PU foam and expands its internal charge storage space, thereby triggering subsequent stronger triboelectricity. Second, this model introduces a novel strategy for the efficient utilization of the volume effect in thick dielectric films, promoting a qualitative leap in FEM-TENG’s performance. The FEM-TENG obtains an average volume power density of 1,591 W m^−3^ Hz^−1^ (average areal power density, 16.061W m^−2^ Hz^−1^) and a charge density of 9.807 mC m^−2^ (volume charge density, 971.6 mC m^−3^), which are significant performance breakthroughs in the field of DC-TENGs. More excitingly, its CF is 1.00375, which demonstrates the constant DC output and sets a new record for TENGs. Finally, the FEM-TENG has ultrahigh durability (100%). Its ability to harvest wind energy (1.8 to 3.6 m s^−1^) has been demonstrated with average power density value as high as 8.461 W m^−2^. This achievement represents a historical milestone for TENGs in the field of breeze energy collection. By collecting breeze energy, the FEM-TENG directly illuminates 3,712 LEDs (no flashing) and continuously drives 50 thermohygrometers. In demonstrations, the 10-cm-diameter FEM-TENG drives 2 lamps (rated power, 2×50 W), a portable pH meter (rated voltage, 6 V), and a Bluetooth thermohygrometer, respectively. This work has achieved model establishment, performance breakthroughs, and practical applications of the DC-TENG, further promoting the commercialization of TENGs.

## Materials and Methods

### Fabrication of the conventional ternary DC-TENG in sliding mode

The conventional ternary DC-TENG in sliding mode mainly consisted of 2 parts: the stator and the rotor. For the slider, (a) a laser cutting machine was used to cut the acrylic plate into a square base plate (55 mm in side length, 3 mm in thickness). Two rectangular notches (48 mm in length, 10 mm in width) were etched onto the square substrate, spaced 15 mm apart. Rectangular notches were chamfered. One of the rectangular notches was 3.75 mm away from the edge of the square substrate. An aluminum foil piece with a thickness of 20 μm was pasted on the acrylic plate and cut along the notches, which serves as one pair of electrodes for the 2-unit traditional sliding ternary DC-TENG. (b) The PU foam was cut to the same size as the acrylic plate and carved with the same notches. The aluminum foil was pasted on the notches of the PU foam as another pair of electrodes. (c) Two pieces of PTFE films (48 mm in length, 10 mm in width, and 50 μm in thickness) were pasted on top of the electrodes on the PU foam as electronegative triboelectric materials. (d) The PU foam and the substrate were pasted together, and the electrodes on the PU foam and the electrodes on the substrate formed a staggered pattern when viewed from above. Finally, the 2 pairs of electrodes were led out by wires in preparation for subsequent testing. More specific details are shown in Fig. S9.

For the stator, (a) the acrylic plate was cut into a rectangular substrate (200 mm in length, 70 mm in width, 3 mm in thickness). (b) A piece of PF was trimmed to match the substrate’s dimensions. (c) Separate holes (5 mm in diameter, 25 mm in distance between holes) were drilled in the substrate to fasten the PF by using tie wraps, making sure that the holes were located approximately 3 mm away from the edge.

### Fabrication of the sliding FEM-TENG

The fabrication process for a sliding FEM-TENG closely mirrored that of the traditional ternary DC-TENG in the sliding mode. The key distinction lay in the incorporation of specially embedded steel needles on the electrode substrate in the case of the FEM-TENG. (a) When a substrate with a side length of 55 mm is engraved with 2 notches (48 mm in length, 10 mm in width), some circular holes were punched inside the rectangular notches by the laser cutting machine. The diameters of the holes ranged from 0.4 to 0.7 mm as required. The spacing between holes varies from 2.5 mm to 5 mm as required. (b) Conductive silver paint was sprayed onto the substrate after masking off areas other than the nicks on the substrate. Conductive silver paint wrapped the steel needles and filled the inside of the notches, as shown in Fig. S3C. (c) After the conductive silver paint dried, the shield was removed and wires were used to connect the 2 electrodes. Then, the other parts of the slider and the stator were made the same as the conventional sliding ternary DC-TENG, which was demonstrated in Fig. S9.

### Fabrication of the rotating FEM-TENG

The rotation mode FEM-TENG mainly includes 2 parts: the stator and the rotor. For the stator: (a) the acrylic sheet was cut into the disc substrate (3 mm in thickness, 10 mm in inner diameter, 110 mm in outer diameter). A central round hole was designed to accommodate the bearing. (b) Eight radial array shapes of uniform size were etched onto the substrate (70 mm in inner diameter, 100 mm in outer diameter). Each radial shape spanned a central angle of 45°, and the edges of the radial arrays were chamfered. Small holes were drilled at intervals of 2.5 mm within the radial array (diameter, 0.7 mm). The steel needles were inserted into the substrate. Conductive silver paint was sprayed into the radial array and wrapped around the steel needles, which then formed the electrodes. (c) The PU foam was cut into discs (2 mm in thickness, 68 mm in inner diameter, 110 mm in outer diameter). Similarly, on the PU foam, 8 uniformly sized radial array shapes were carved, and aluminum foil was affixed onto the radial array as another set of output electrodes. (d) The 8 radial arrays were covered with PTFE films of the same size as electronegative triboelectric materials. Finally, the substrate and the PU foam were combined to form a complete rotating FEM-TENG. The total triboelectric layer area of the rotating FEM-TENG is 33.175 cm^2^.

For the rotator: (a) the acrylic sheet was cut into the disc (3 mm in thickness, 10 mm in inner diameter, 180 mm in outer diameter), serving as the substrate. (b) A piece of PF matching the dimensions of the substrate was cut. (c) Holes were drilled 15 mm away from the edge of the acrylic sheet (diameter, 5 mm), and the PF was fixed to the acrylic sheet with the ties.

### Electric measurement and characterization

The sliding motion of the slide blocks of the FEM-TENG and the traditional ternary DC-TENG in the sliding modes was powered by the linear motor (LinMot PS01-37×120-C). The FEM-TENG’s rotator was powered by a commercial programmable stepping motor (86HSE12N). A wind system consisted of a simple wind cup and a centrifugal fan designed to simulate natural wind conditions. The wind cup blades were propelled by a centrifugal fan, and the wind speed was regulated by adjusting the input voltage. A high-voltage power supply (DW-N303-1ACH2) was used to apply DC voltage ranging from 0 V to 3,000 V to the PU foam, while simultaneously testing its leakage current. An electrometer (Keithley 6514) and a high-speed electrostatic voltmeter (Trek model 370) were used to record the output charge, current, and voltage. A SEM (Quattro S, Thermo Fisher Scientific) was used to characterize the surface morphology of triboelectric materials before and after the stability test. PF and steel needles were purchased from the store. The digital force gauge for testing friction force fixed on the slide is SL-50. The test environment temperature was 26°Celsius and the humidity is 70%. Safety risk assessment of embedded needle electrodes is shown in Note S4.

## Data Availability

The data that support the findings of this study are available from the corresponding author upon reasonable request.

## Supplementary Materials

Supplementary 1 Materials and Methods Figs. S1 to S17 Tables S1 to S13 Notes S1 to S4 Movies S1 to S5

## References

[1] Ren G, Hu Q, Ye J, Hu A, Lü J, Zhou S. All-biobased hydrovoltaic-photovoltaic electricity generators for all-weather energy harvesting. Research. 2022;2022: Article 9873203. 36082209 10.34133/2022/9873203 PMC9429978

[2] Liu R, Wang ZL, Fukuda K, Someya T. Flexible self-charging power sources. Nat Rev Mater. 2022;7:870–886.

[3] Pu X, Guo H, Chen J, Wang X, Xi Y, Hu C, Wang ZL. Eye motion triggered self-powered mechnosensational communication system using triboelectric nanogenerator. Sci Adv. 2017;3: Article e1700694. 28782029 10.1126/sciadv.1700694 PMC5533541

[4] Chen J, Wu K, Gong S, Wang J, Wang K, Guo H. A magnetic-multiplier-enabled hybrid generator with frequency division operation and high energy utilization efficiency. Research. 2023;6: Article 0168. 37303603 10.34133/research.0168 PMC10254463

[5] He W, Liu W, Fu S, Wu H, Shan C, Wang Z, Xi Y, Wang X, Guo H, Liu H, et al. Ultrahigh performance triboelectric nanogenerator enabled by charge transmission in interfacial lubrication and potential decentralization design. Research. 2022;2022:9812865. 35909938 10.34133/2022/9812865 PMC9285635

[6] Peng X, Dong K, Ye C, Jiang Y, Zhai S, Cheng R, Liu D, Gao X, Wang J, Wang ZL. A breathable, biodegradable, antibacterial, and self-powered electronic skin based on all-nanofiber triboelectric nanogenerators. Sci Adv. 2020;6(26):eaba9624. 32637619 10.1126/sciadv.aba9624 PMC7319766

[7] Wang H, Wang J, Yao K, Fu J, Xia X, Zhang R, Li J, Xu G, Wang L, Yang J, et al. A paradigm shift fully self-powered long-distance wireless sensing solution enabled by discharge-induced displacement current. Sci Adv. 2021;7(39):eabi6751. 34550743 10.1126/sciadv.abi6751 PMC8457664

[8] Yang H, Lai J, Li Q, Zhang X, Li X, Yang Q, Hu Y, Xi Y, Wang ZL. High-sensitive and ultra-wide spectrum multifunctional triboelectric acoustic sensor for broad scenario applications. Nano Energy. 2022;104(Part A): Article 107932.

[9] Zu L, Wen J, Wang S, Zhang M, Sun W, Chen B, Wang ZL. Multiangle, self-powered sensor array for monitoring head impacts. Sci Adv. 2023;9(20):eadg5152. 37196075 10.1126/sciadv.adg5152 PMC10191426

[10] Li Q, Liu W, Yang H, He W, Long L, Wu M, Zhang X, Xi Y, Hu C, Wang ZL. Ultra-stability high-voltage triboelectric nanogenerator designed by ternary dielectric triboelectrification with partial soft-contact and non-contact mode. Nano Energy. 2021;90(Part B): Article 106586.

[11] Zhang X, Yang Q, Ji P, Wu Z, Li Q, Yang H, Li X, Zheng G, Xi Y, Wang ZL. Modeling of liquid-solid hydrodynamic water wave energy harvesting system based on triboelectric nanogenerator. Nano Energy. 2022;99: Article 107362.

[12] Ren D, Yang H, Zhang X, Li Q, Yang Q, Li X, Ji P, Yu P, Xi Y, Wang ZL. Ultra-high DC and low impedance output for free-standing triboelectric nanogenerator. Adv Energy Mater. 2023;13:2302877.

[13] Yang P, Shi Y, Tao X, Liu Z, Dong X, Wang ZL, Chen X. Radical anion transfer during contact electrification and its compensation for charge loss in triboelectric nanogenerator. Matter. 2023;6:1295–1311.

[14] Tao X, Yang P, Liu Z, Qin S, Hu J, Huang ZX, Chen X, Qu JP. Acid-doped pyridine-based polybenzimidazole as a positive triboelectric material with superior charge retention capability. ACS Nano. 2024;18:4467–4477. 38263634 10.1021/acsnano.3c11087

[15] Qin S, Chen J, Yang P, Liu Z, Tao X, Dong X, Hu J, Chu X, Wang ZL, Chen X. A piezo-tribovoltaic nanogenerator with ultrahigh output power density and dynamic sensory functions. Adv Energy Mater. 2024;14(12):2303080.

[16] Tang Y, Xu B, Tan D, Han J, Gao Y, Li Z, Liu X. Ultrastrong-polar cyano-prussian blue analogs hybrid tribomaterials for biomechanical energy harvesting and self-powered sensing. Nano Energy. 2023;110: Article 108358.

[17] Li Z, Xu B, Han J, Tan D, Huang J, Gao Y, Fu H. Surface-modified liquid metal nanocapsules derived multiple triboelectric composites for efficient energy harvesting and wearable self-powered sensing. Chem Eng J. 2023;460: Article 141737.

[18] Tang Y, Xu B, Gao Y, Li Z, Tan D, Li M, Liu Y, Huang J. Ultrastrong-polar polyacrylonitrile organic-inorganic architected nanogenerators with synergistic triboelectric behavior for efficient biomechanical energy harvesting and self-powered sensing. Nano Energy. 2022;103: Article 107833.

[19] Gong J, Xu B, Yang Y, Wu M, Yang B. An adhesive surface enables high-performance mechanical energy harvesting with unique frequency-insensitive and pressure-enhanced output characteristics. Adv Mater. 2020;32(14):1907948. 10.1002/adma.201907948 32080915

[20] Li Z, Xu B, Han J, Huang J, Chung KY. Interfacial polarization and dual charge transfer induced high permittivity of carbon dots-based composite as humidity-resistant tribomaterial for efficient biomechanical energy harvesting. Adv Energy Mater. 2021;11(30):2101294.

[21] Wang X, Song J, Liu J, Wang ZL. Direct-current nanogenerator driven by ultrasonic waves. Science. 2007;316(5821):102–105. 17412957 10.1126/science.1139366

[22] Li Q, Fu S, Li X, Chen H, He W, Yang Q, Zhang X, Yang H, Ren D, Xi Y. Overall performance improvement of direct-current triboelectric nanogenerators by charge leakage and ternary dielectric evaluation. Energy Environ Sci. 2023;16:3514–3525.

[23] Liu D, Yin X, Guo H, Zhou L, Li X, Zhang C, Wang J, Wang ZL. A constant current triboelectric nanogenerator arising from electrostatic breakdown. Sci Adv. 2019;5(4):eaav6437. 30972365 10.1126/sciadv.aav6437 PMC6450689

[24] Zhao Z, Dai Y, Liu D, Zhou L, Li S, Wang ZL, Wang J. Rationally patterned electrode of direct-current triboelectric nanogenerators for ultrahigh effective surface charge density. Nat Commun. 2020;11:6186. 33273477 10.1038/s41467-020-20045-y PMC7712892

[25] Li X, Zhang C, Gao Y, Zhao Z, Hu Y, Yang O, Liu L, Zhou L, Wang J, Wang ZL. A highly efficient constant-voltage triboelectric nanogenerator. Energy Environ Sci. 2022;15:1334–1345.

[26] Li K, Shan C, Fu S, Wu H, He W, Wang J, Li G, Mu Q, du S, Zhao Q, et al. High efficiency triboelectric charge capture for high output direct current electricity. Energy Environ Sci. 2024;17:580–590.

[27] Zhang Z, Wang Z, Chen Y, Feng Y, Dong S, Zhou H, Wang ZL, Zhang C. Semiconductor contact-electrification-dominated tribovoltaic effect for ultrahigh power generation. Adv Mater. 2022;34(20):2200146. 10.1002/adma.202200146 35291054

[28] Li Q, Hu Y, Yang Q, Li X, Zhang X, Yang H, Ji P, Xi Y, Wang ZL. A robust constant-voltage DC triboelectric nanogenerator using the ternary dielectric triboelectrification effect. Adv Energy Mater. 2023;13(2):2202921.

[29] Wu Z, Wang S, Cao Z, Ding R, Ye X. Rotary disk multi-phase freestanding-electret generator with enhanced power and low ripple output. Nano Energy. 2021;83: Article 105787.

[30] Chen P, An J, Cheng R, Shu S, Berbille A, Jiang T, Wang ZL. Rationally segmented triboelectric nanogenerator with a constant direct-current output and low crest factor. Energy Environ Sci. 2021;14:4523–4532.

[31] Zhou L, Liu D, Li S, Zhao Z, Zhang C, Yin X, Liu L, Cui S, Wang ZL, Wang J. Rationally designed dual-mode triboelectric nanogenerator for harvesting mechanical energy by both electrostatic induction and dielectric breakdown effects. Adv Energy Mater. 2020;10(24):2000965.

[32] Zeng Q, Chen A, Zhang X, Luo Y, Tan L, Wang X. A dual-functional triboelectric nanogenerator based on the comprehensive integration and synergetic utilization of triboelectrification, electrostatic induction, and electrostatic discharge to achieve alternating current/direct current convertible outputs. Adv Mater. 2023;35(7):2208139. 10.1002/adma.202208139 36349825

[33] Wu H, Wang S, Wang Z, Zi Y. Achieving ultrahigh instantaneous power density of 10 MW/m^-2^ by leveraging the opposite-charge-enhanced transistor-like triboelectric nanogenerator (OCT-TENG). Nat Commun. 2021;12:5470. 34526498 10.1038/s41467-021-25753-7 PMC8443631

[34] Wu H, Wang J, Fu S, Shan C, Zhao Q, Li K, Li G, Mu Q, Wang X, Hu C. A constant current triboelectric nanogenerator achieved by hysteretic and ordered charge migration in dielectric polymers. Energy Environ Sci. 2023;16:5144–5153.

[35] Fu S, Wu H, He W, Li Q, Shan C, Wang J, du Y, du S, Huang Z, Hu C. Conversion of dielectric surface effect into volume effect for high output energy. Adv Mater. 2023;35(40):2302954. 10.1002/adma.202302954 37354126

[36] Shan C, Li K, Wu H, Fu S, Liu A, Wang J, Zhang X, Liu B, Wang X, Hu C. Ternary electrification for efficient charge accumulation and synergetic charge collection. Adv Funct Mater. 2024;34:2310332.

[37] Lai M, Du B, Guo H, Xi Y, Yang H, Hu C, Wang J, Wang ZL. Enhancing the output charge density of TENG via building longitudinal paths of electrostatic charges in the contacting layers. ACS Appl Mater Interfaces. 2018;10(2):2158–2165. 29261275 10.1021/acsami.7b15238

[38] Liu Z, Nie J, Miao B, Li J, Cui Y, Wang S, Zhang X, Zhao G, Deng Y, Wu Y, et al. Self-powered intracellular drug delivery by a biomechanical energy-driven triboelectric nanogenerator. Adv Mater. 2019;31(12):1807795. 10.1002/adma.201807795 30721538

[39] Shi L, Li Z, Chen M, Zhu T, Wu L. Ultrasensitive and ultraprecise pressure sensors for soft systems. Adv Mater. 2023;35(10):2210091. 10.1002/adma.202210091 36625165

[40] Yu Q, Ge R, Wen J, du T, Zhai J, Liu S, Wang L, Qin Y. Highly sensitive strain sensors based on piezotronic tunneling junction. Nat Commun. 2022;13:778. 35140219 10.1038/s41467-022-28443-0 PMC8828782

[41] Zhao H, Wang H, Yu H, Xu Q, Li X, Guo J, Shao J, Wang ZL, Xu M, Ding W. Theoretical modeling of contact-separation mode triboelectric nanogenerators from initial charge distribution. Energy Environ Sci. 2024;17:2228–2247.

[42] Ryu H, Lee J, Khan U, Kwak SS, Hinchet R, Kim SW. Sustainable direct current powering a triboelectric nanogenerator: Via a novel asymmetrical design. Energy Environ Sci. 2018;11(8):2057–2063.

[43] Wang Z, Zhang Z, Chen Y, Gong L, Dong S, Zhou H, Lin Y, Lv Y, Liu G, Zhang C. Achieving an ultrahigh direct-current voltage of 130 V by semiconductor heterojunction power generation based on the tribovoltaic effect. Energy Environ Sci. 2022;15:2366–2373.

[44] Du Y, Fu S, Shan C, Wu H, He W, Wang J, Guo H, Li G, Wang Z, Hu C. A novel design based on mechanical time-delay switch and charge space accumulation for high output performance direct-current triboelectric nanogenerator. Adv Funct Mater. 2022;32(48):2208783.

[45] Pang H, Feng Y, An J, Chen P, Han J, Jiang T, Wang ZL. Segmented swing-structured fur-based triboelectric nanogenerator for harvesting blue energy toward marine environmental applications. Adv Funct Mater. 2021;31(47):2106398.

[46] Yong S, Wang H, Lin Z, Li X, Zhu B, Yang L, Ding W, Liao R, Wang J, Wang ZL. Environmental self-adaptive wind energy harvesting technology for self-powered system by triboelectric-electromagnetic hybridized nanogenerator with dual-channel power management topology. Adv Energy Mater. 2022;12(43):2202469.

[47] You Z, Wang X, Lu F, Wang S, Hu B, Li L, Fang W, Liu Y. An organic semiconductor/metal Schottky heterojunction based direct current triboelectric nanogenerator windmill for wind energy harvesting. Nano Energy. 2023;109: Article 108302.

[48] Han J, Feng Y, Chen P, Liang X, Pang H, Jiang T, Wang ZL. Wind-driven soft-contact rotary triboelectric nanogenerator based on rabbit fur with high performance and durability for smart farming. Adv Funct Mater. 2022;32(2):2108580.

[49] Zhang C, Liu Y, Zhang B, Yang O, Yuan W, He L, Wei X, Wang J, Wang ZL. Harvesting wind energy by a triboelectric nanogenerator for an intelligent high-speed train system. ACS Energy Lett. 2021;6(4):1490–1499.

[50] He W, Shan C, Fu S, Wu H, Wang J, Mu Q, Li G, Hu C. Large harvested energy by self-excited liquid suspension triboelectric nanogenerator with optimized charge transportation behavior. Adv Mater. 2023;35(7):2209657. 10.1002/adma.202209657 36398558

[51] Mu Q, He W, Shan C, Fu S, Du S, Wang J, Wang Z, Li K, Hu C. Achieving high-efficiency wind energy harvesting triboelectric nanogenerator by coupling soft contact, charge space accumulation, and charge dissipation design. Adv Funct Mater. 2023;34(2):2309421.

[52] Long L, Liu W, Wang Z, He W, Li G, Tang Q, Guo H, Pu X, Liu Y, Hu C. High performance floating self-excited sliding triboelectric nanogenerator for micro mechanical energy harvesting. Nat Commun. 2021;12:4689. 34344899 10.1038/s41467-021-25047-y PMC8333367

[53] Ji P, Li Q, Zhang X, Hu Y, Han X, Zhang D, Hu C, Xi Y. Achieving continuous self-powered energy conversion-storage-supply integrated system based on carbon felt. Adv Sci. 2023;10(13):2207033. 10.1002/advs.202207033 PMC10161012 36876443

