# Supplementary material for: Achieving Ultrahigh DC-Power Triboelectric Nanogenerators by Lightning Rod-Inspired Field Emission Modeling
Source: Research (Wash D C). 2024 Aug 13;7:0437. doi: 10.34133/research.0437 (PMC11320116; doi:10.34133/research.0437)
Supplement: Supplementary 1 — Materials and Methods Figs. S1 to S17 Tables S1 to S13 Notes S1 to S4 Movies S1 to S5 [file research.0437.f1.zip › Reviced Supplementary Information.pdf]

Supporting Information for

Achieving Ultrahigh DC-Power Triboelectric Nanogenerator by Lightning Rod-Inspired Field Emission Modeling

Qianying Li[1,†], Shaoke Fu[1,†], Huake Yang[1], Xiaochuan Li[1], Xuemei Zhang[1], Chenguo Hu[1], and Yi Xi[1,*]

[1] Department of Applied Physics, Chongqing Key Laboratory of Materials Physics, College of Physics, Chongqing University, Chongqing, 400044, P. R. China.

† *These authors contributed equally to this work.*

* Correspondence should be addressed to Yi Xi; yxi6@cqu.edu.cn.

Contents

Figure S1. Schematic diagram of the lightning rod.

Figure S2. COMSOL simulation parameters of FEM-TENG and ternary DC-TENG.

Figure S3. Photograph of 2-unit FEM-TENG.

Figure S4. Two-dimensional schematic diagram and output characteristics of sliding FEM-TENG.

Figure S5. Working mechanism of the sliding FEM-TENG before reaching charge saturation state.

Figure S6. Detailed working mechanism of sliding FEM-TENG after reaching charge saturation state.

Figure S7. Analysis on the working mechanism of FEM-TENG and ordinary ternary DC-TENG.

Figure S8. Output of conventional ternary DC-TENGs with different thicknesses of PU foam.

Figure S9. Manufacturing process of the slider of sliding DC-TENGs.

Figure S10. Performance of FEM-TENG after inserting needles of different diameters.

Figure S11. Performance of sliding FEM-TENG with different needle densities.

Figure S12. Voltage and frictional force of sliding FEM-TENG.

Figure S13. Photographs of rotating FEM-TENG.

Figure S14. DC charge output of the FEM-TENG at 60 rpm after rotating 0.5 revolutions.

Figure S15. Durability test of the rotating FEM-TENG.

Figure S16. Output performance of FEM-TENG under different wind speeds.

Figure S17. Voltage curves for charging of different capacitors when FEM-TENG with PMC is driven by wind.

Table S1. Comparison of average power density and crest factor with the latest and most typical DC-TENGs.

Table S2. The detailed motion parameters of sliding FEM-TENG.

Table S3. Voltage crest factor of sliding FEM-TENG at different sliding speeds.

Table S4. Voltage crest factor of rotating FEM-TENG at different rotating speeds.

Table S5. Current crest factor of rotating FEM-TENG at different rotating speeds.

Table S6. Comparison of charge density per round with the latest and most typical rotating DC-TENGs.

Table S7. Comparison of energy conversion efficiency with the most representative rotary TENG.

Table S8. Voltage crest factor of FEM-TENG before and after the stability test.

Table S9. Wind speed under different input voltages.

Table S10. Current crest factor of FEM-TENG under different wind speeds.

Table S11. Voltage crest factor of FEM-TENG under different wind speeds.

Table S12. Power density comparison with state-of-the-art rotary wind-driven TENGs.

Table S13. Charging rates of various capacitors when FEM-TENG is driven by wind and stepper motors respectively.

Note S1. Working mechanism of the sliding FEM-TENG before reaching charge saturation state.

Note S2. The calculation of friction coefficient.

Note S3. Energy conversion efficiency of FEM-TENG.

Note S4 Safety risks posed by needle-embedded back electrodes.

References

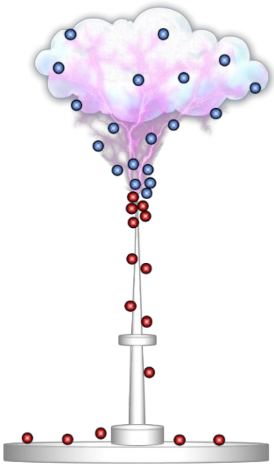

Figure S1. Schematic diagram of the lightning rod.

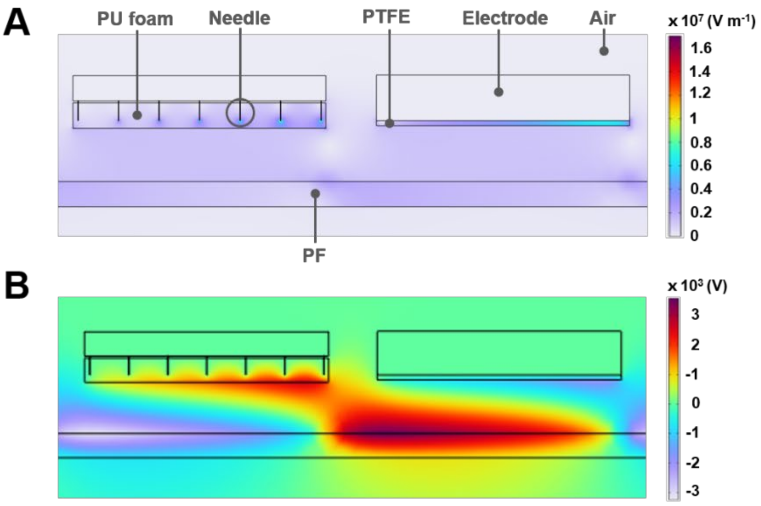

C

Parameters for simulation	
PTFE	Thickness: 0.01 mm; width: 0.5 mm; permittivity: 4<br>Surface charge density (FEM-TENG): -0.0005 C m^{-2}<br>Surface charge density (ternary DC-TENG): -0.00008 C m^{-2}
PU foam	Thickness: 0.05 mm; width: 0.5 mm; permittivity: 6<br>Surface charge density (FEM-TENG): 0.0005 C m^{-2}<br>Surface charge density (ternary DC-TENG): 0.00008 C m^{-2}
PF	Thickness: 0.05 mm; width: 0.5 mm<br>Permittivity: 3<br>Surface charge density (below the PU foam, FEM-TENG): -0.0005 C m^{-2}<br>Surface charge density (below the PTFE, FEM-TENG): 0.0005 C m^{-2}<br>Surface charge density (below the PU foam, ternary DC-TENG): -0.00008 C m^{-2}<br>Surface charge density (below the PU foam, ternary DC-TENG): 0.00008 C m^{-2}
Electrode	Thickness: 0.05 mm; width: 0.5 mm (located on the back of PU foam)<br>Thickness: 0.09 mm; width: 0.5 mm (located on the back of PTFE)<br>Permittivity: 99999
Needle	Diameter: 0.001 mm; length: 0.04 mm<br>Permittivity: 99999

Figure S2. COMSOL simulation parameters of FEM-TENG and ternary DC-TENG. (A) Electric field strength between the triboelectric films of FEM-TENG. (B) Potential distribution of FEM-TENG. (B) Simulation parameters related to each component.

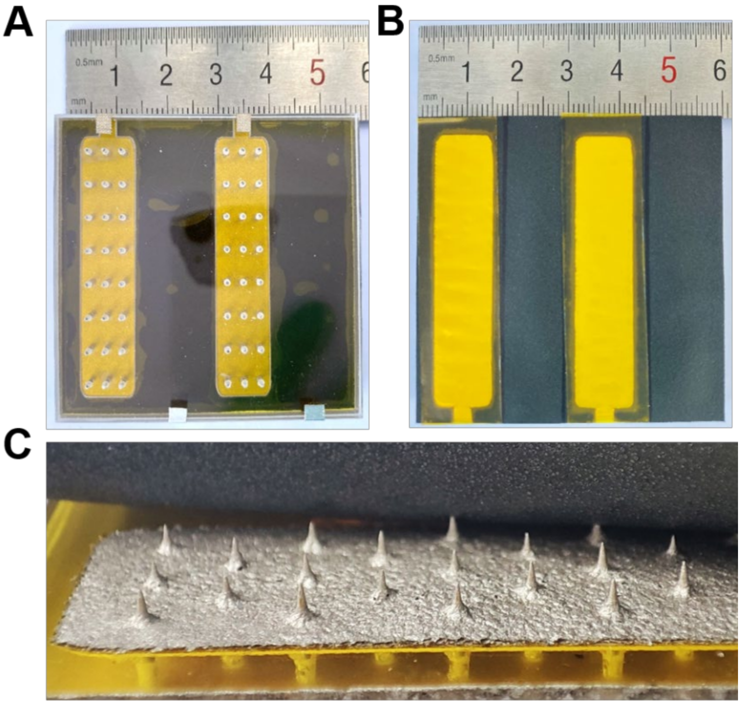

Figure S3. Photograph of 2-unit FEM-TENG. (A) Back base view. (B) Top view of 2-unit FEE-TENG. (C) Enlarged view of the electrodes with needles.

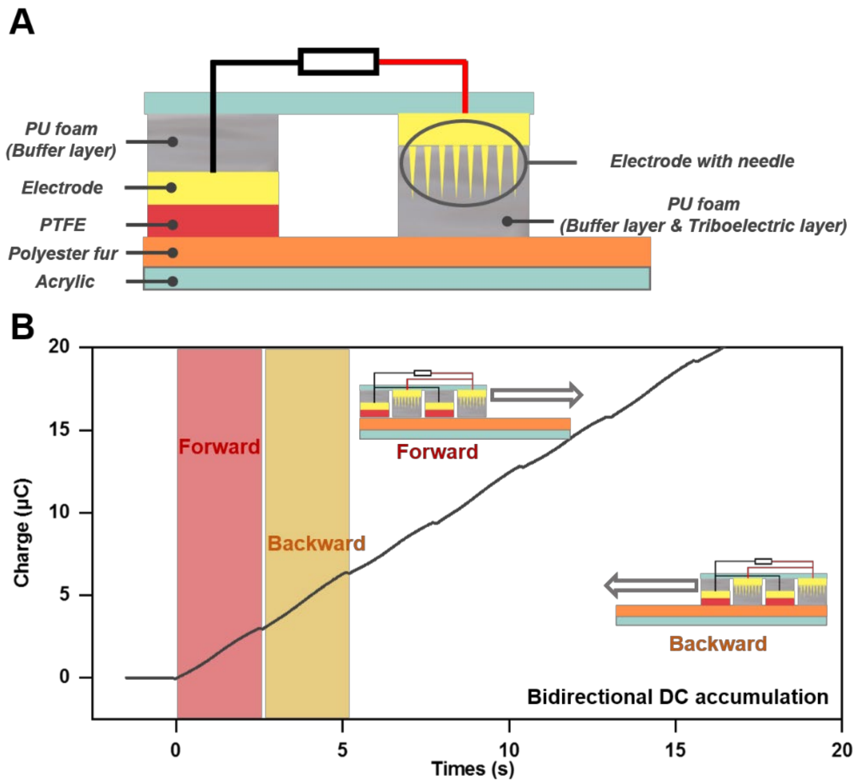

Figure S4. Two-dimensional schematic diagram and output characteristics of sliding FEM-TENG. (A) Two-dimensional schematic diagram of the 1-unit sliding FEM-TENG and an introduction to each part. (B) Charge output of the sliding FEM-TENG with bidirectional DC charge accumulation in reciprocating motions.

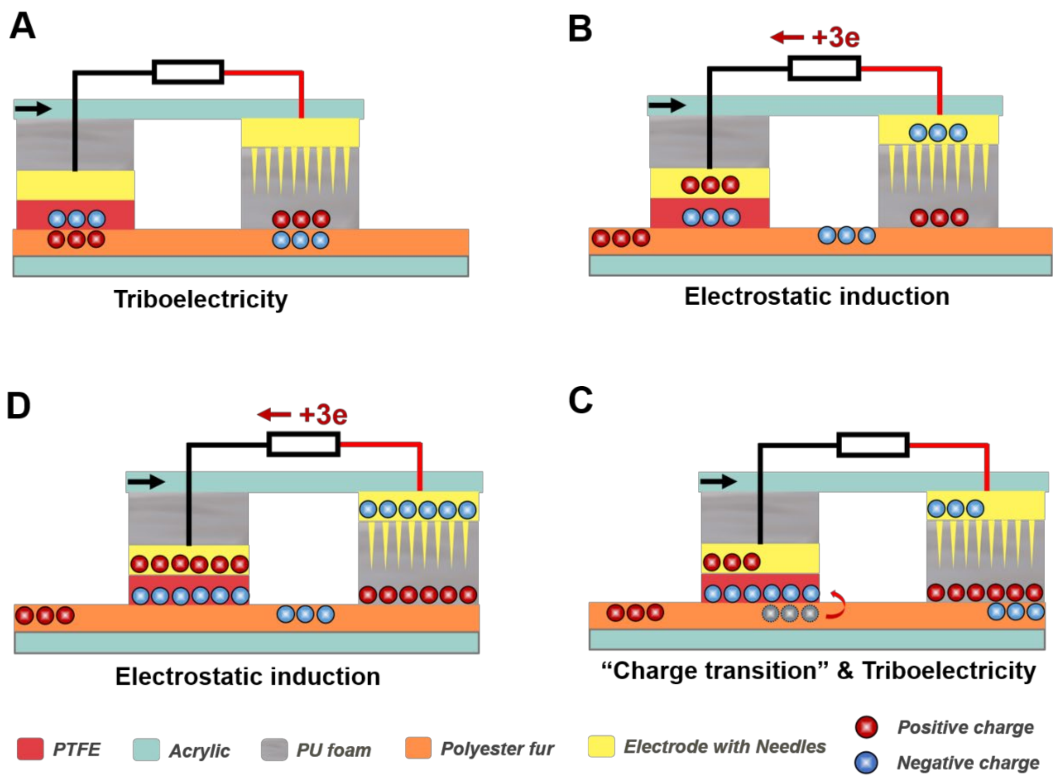

Figure S5. Working mechanism of the sliding FEM-TENG before reaching charge saturation state.

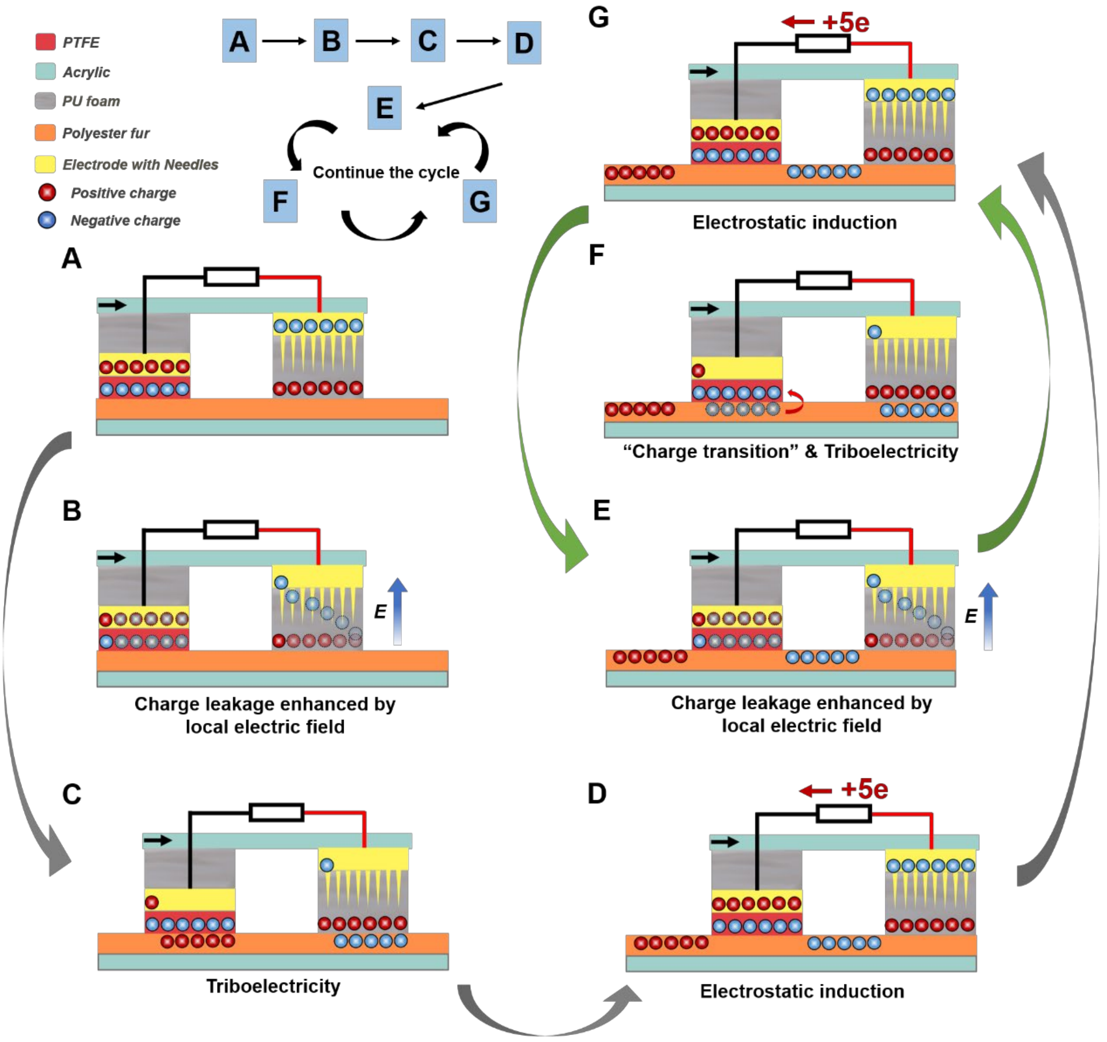

Figure S6. Detailed working mechanism of sliding FEM-TENG after reaching charge saturation state.

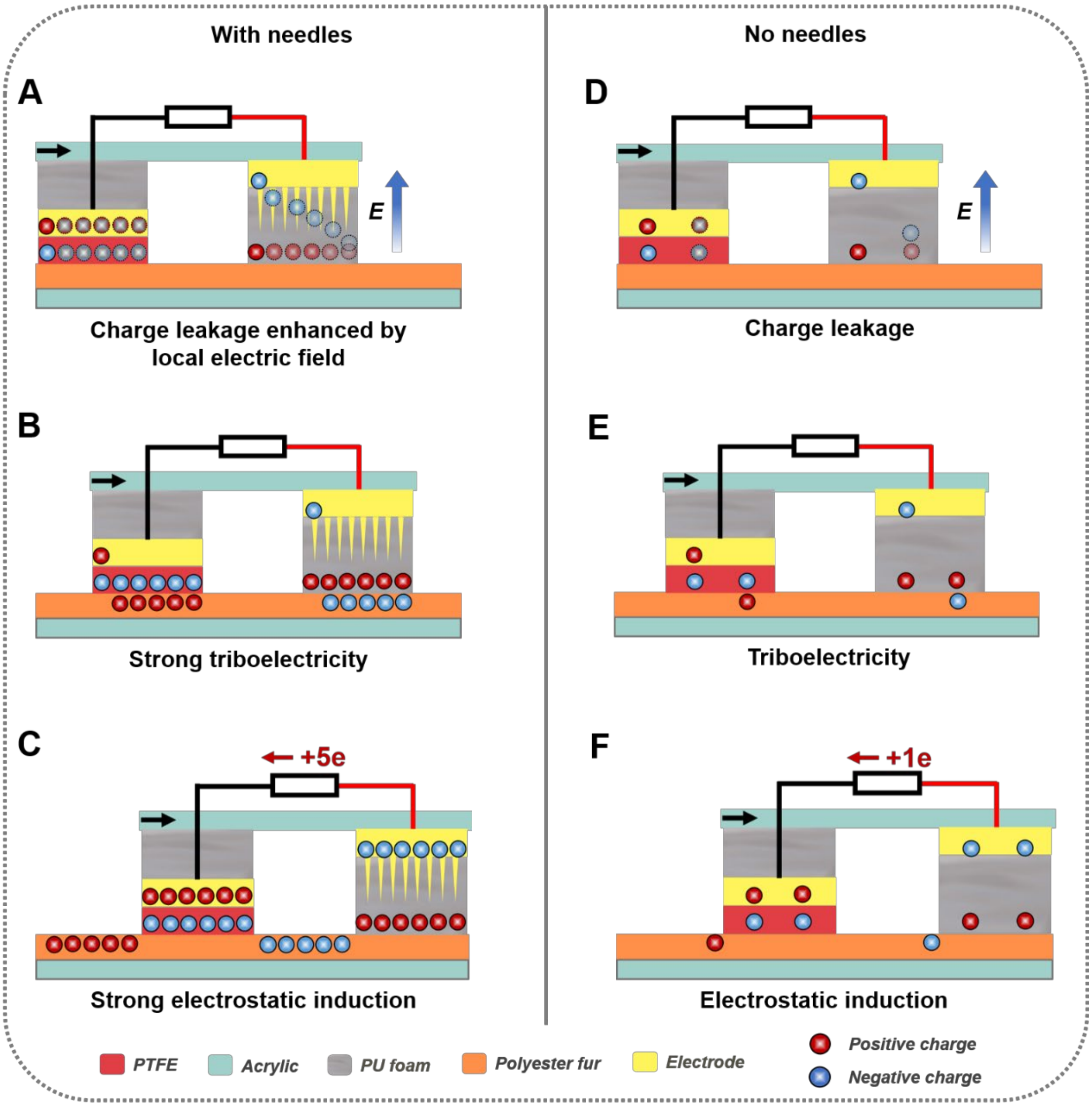

Figure S7. Analysis on the working mechanism of FEM-TENG and ordinary ternary DC-TENG. (A to C) Working mechanism of FEM-TENG. (D to F) working mechanism of ordinary ternary DC-TENG.

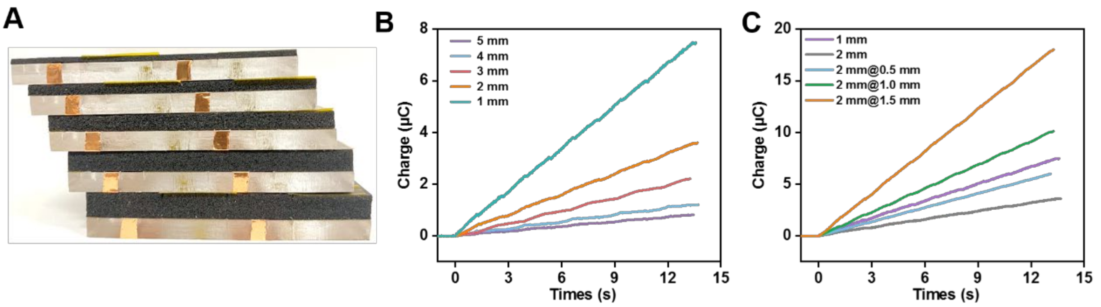

Figure S8. Output of conventional ternary DC-TENGs with different thicknesses of PU foam. (A) Photograph of conventional ternary DC-TENGs with different thicknesses of PU foam. (B) Original charge data diagram of conventional ternary DC-TENGs with PU foam of different thicknesses. (C) Original charge data diagram of FEM-TENG after inserting needles of different lengths.

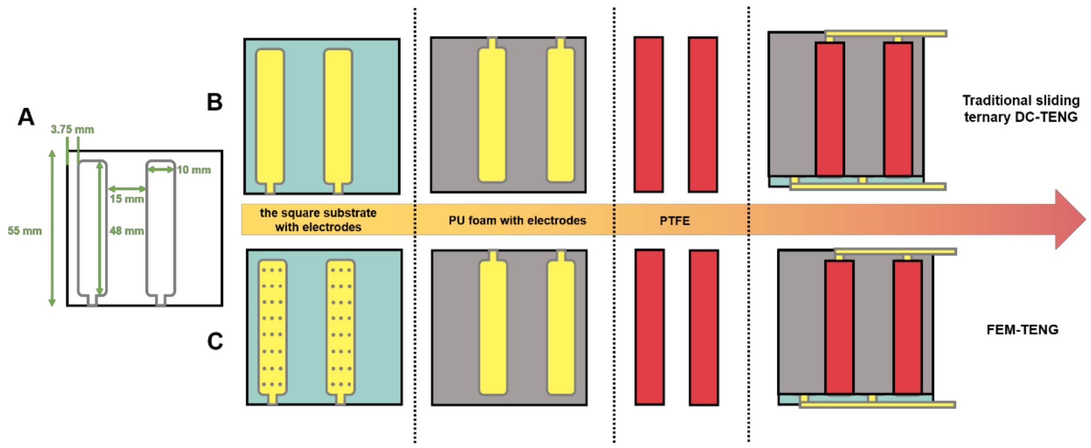

Figure S9. Manufacturing process of the slider of sliding DC-TENGs. (A) Slider substrate size of 2-unit DC-TENG. (B) Manufacturing process of the slider of the traditional sliding ternary DC-TENG. (C) Manufacturing process of the slider of the FEM-TENG.

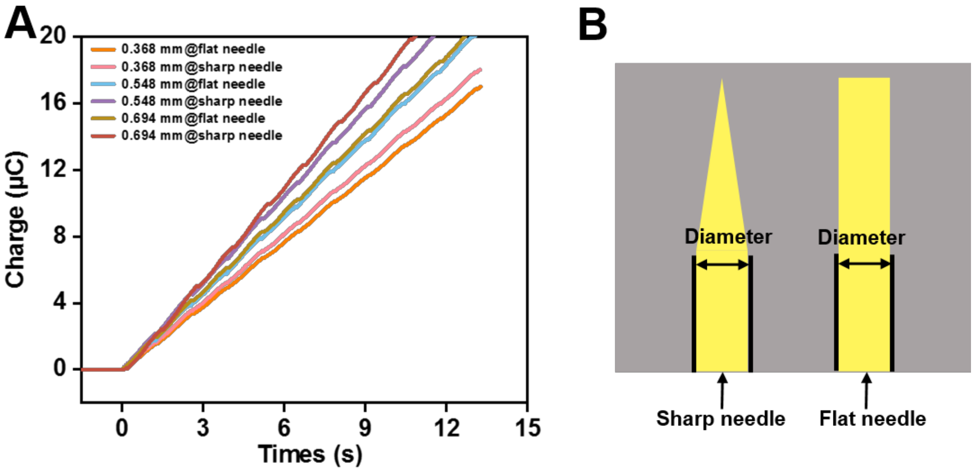

Figure S10. Performance of FEM-TENG after inserting needles of different diameters. (A) Original charge data diagram of FEM-TENG after inserting needles of different diameters. (B) Schematic diagram of needles.

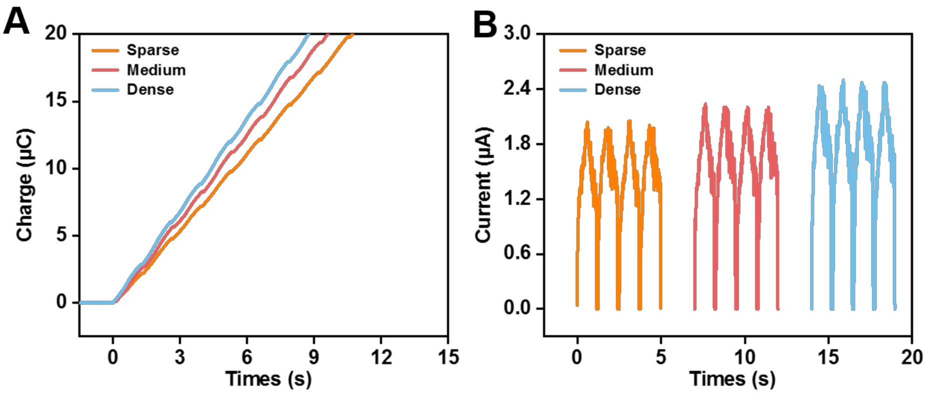

Figure S11. Performance of sliding FEM-TENG with different needle densities. (A) Original charge data diagram of FEM-TENG with different needle densities. (B) Original current data diagram of FEM-TENG with different needle densities.

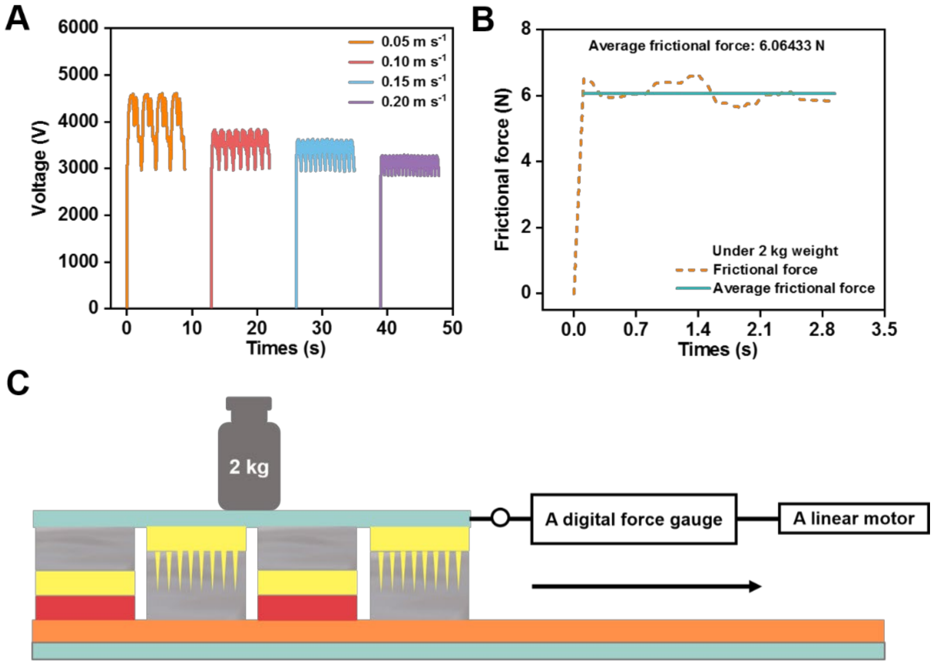

Figure S12. Voltage and frictional force of sliding FEM-TENG. (A) Voltage of sliding FEM-TENG at different sliding speeds. (B) Frictional force and average frictional force between the slider and stator (polyester fur) of a 2-unit FEM-TENG under the pressure of 2 kg weight. (C) Schematic diagram of testing the frictional force.

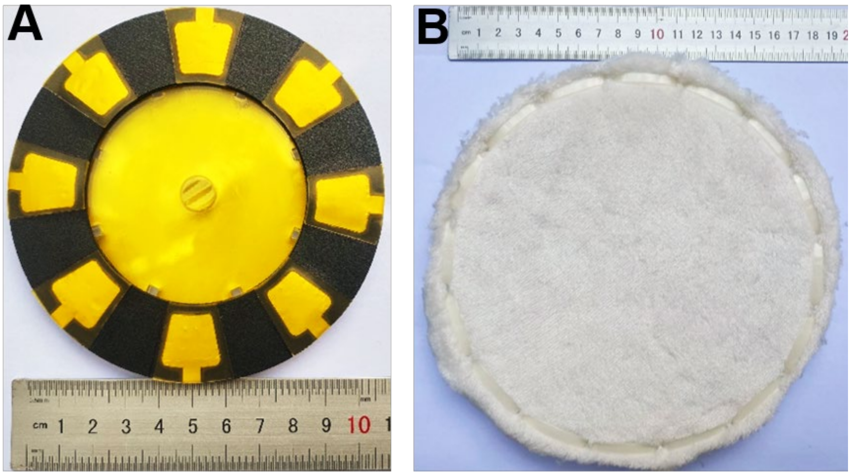

Figure S13. Photographs of rotating FEM-TENG. (A) Photograph of the stator of the rotary FEM-TENG. (B) Photograph of the rotor of the rotating FEM-TENG made of polyester fur.

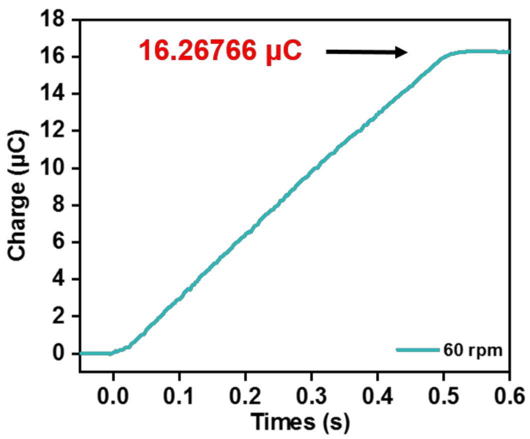

Figure S14. DC charge output of the FEM-TENG at 60 rpm after rotating 0.5 revolutions.

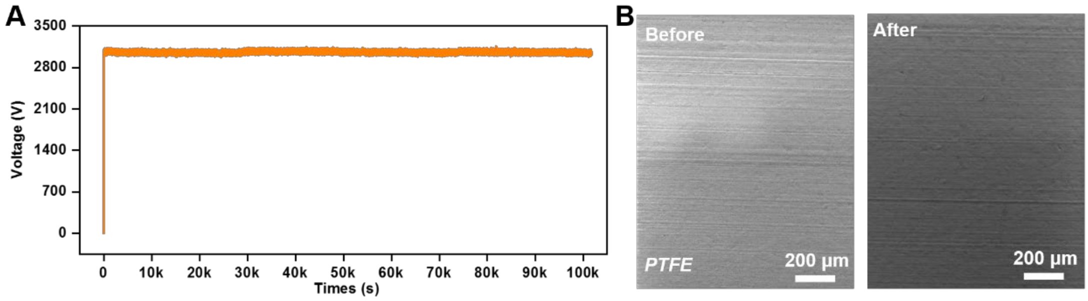

Figure S15. Durability test of the rotating FEM-TENG. (A) Original data chart of 100k cycle test of rotating FEM-TENG. (B) SEM characterization of PTFE films before and after FEM-TENG durability testing.

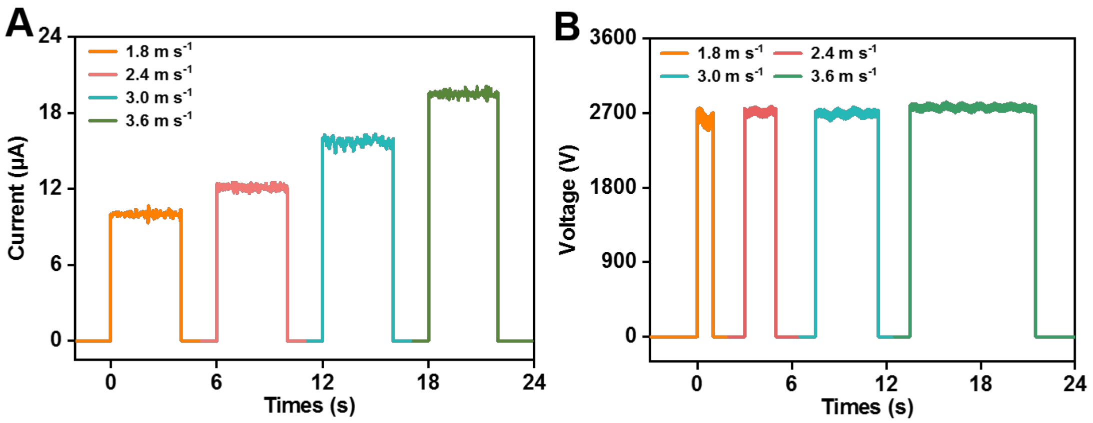

Figure S16. Output performance of FEM-TENG under different wind speeds. (A) Original current data diagram of FEM-TENG under different wind speeds. (B) Original voltage data diagram of FEM-TENG under different wind speeds.

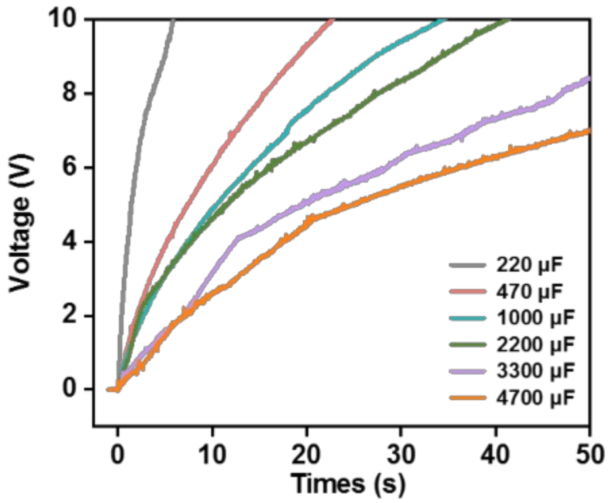

Figure S17. Voltage curves for charging of different capacitors when FEM-TENG with PMC is driven by wind.

Table S1. Comparison of average power density and crest factor with the latest and most typical DC-TENGs [1-14].

Article (DC-TENG)	Type	Materials	Crest factor	Frequency	Volume and area of triboelectric layer	Average power density
Zhou et al *Adv. Energy Mater.* (2020)[1]	Electrostatic breakdown	FEP/Cu	1.2	0.66 Hz	153.86 cm^2<br>152.74 cm^3	0.01072 W m^{-2} Hz^{-1}<br>1.064 W m^{-3} Hz^{-1}
Wu et al *Nano Energy* (2021)[2]	Phase control	PTFE/Cu	1.09	15.8	---	1.225 W m^{-2}<br>0.078 W m^{-2} Hz^{-1}
Li et al *Energy Environ. Sci.* (2022)[3]	Phase control	PVC/Cu	1.03	8 Hz	706.5 cm^2<br>706.5 cm^3	2.032 W m^{-2}<br>0.254 W m^{-2} Hz^{-1}<br>25.4 W m^{-3} Hz^{-1}
Ryu et al *Energy Environ. Sci.* (2018)[4]	Phase control	PTFE/PA	1.26	15.3 Hz	---	4.9 W m^{-2}<br>0.32 W m^{-2} Hz^{-1}
Zeng et al *Adv. Mater.* (2023)[5]	Electrostatic breakdown	PTFE/PU foam	1.1	1 Hz	157 cm^2<br>64.527 cm^3	0.398 W m^{-2} Hz^{-1}<br>96.84 W m^{-3} Hz^{-1}
Wu et al *Nat. Commun.* (2021)[6]	Opposite-charge-enhancement effect	FEP/PC	---	1 Hz	25 cm^2<br>8.25 cm^3	0.79 W m^{-2}<br>0.79 W m^{-2} Hz^{-1}<br>239.39 W m^{-2} Hz^{-1}
Chen et al *Energy Environ. Sci.* (2021)[7]	Phase control	PTFE/fur	1.05	1 Hz	113 cm^2<br>170.8 cm^3	1.355 W m^{-2}<br>1.355 W m^{-2} Hz^{-1}<br>89.65 W m^{-3} Hz^{-1}
Wang et al *Energy Environ. Sci.* (2022)[8]	Tribovoltaic effect	GaN/Si	---	---	---	1.5 W m^{-2}
Zhang et al *Adv. Mater.* (2022)[9]	Tribovoltaic effect	GaN/ Bi_2Te_3	---	6.25 Hz	1.33 cm^2	9.23 W m^{-2}<br>1.48 W m^{-2} Hz^{-1}
Du et al *Adv. Funct. Mater.* (2022)[10]	Mechanical time-delay switch	FEP/PA	1.492	1 Hz	96 cm^2<br>~96 cm^3	2.1 W m^{-2}<br>2.1 W m^{-2} Hz^{-1}<br>~210 W m^{-3} Hz^{-1}
Li et al *Adv. Energy Mater.* (2023)[11]	Ternary dielectric triboelectrification effect	PTFE/ PF/PA	1.0082	2.5 Hz	25.655 cm^2<br>25.835 cm^3	8.77 W m^{-2}<br>3.51 W m^{-2} Hz^{-1}<br>348.56 W m^{-3} Hz^{-1}
Li et al *Energy Environ. Sci.* (2023)[12]	Ternary dielectric triboelectrification effect	PTFE/ PET/PA	1.0043	2 Hz	26.267 cm^2<br>26.582 cm^2	12.3 W m^{-2}<br>6.15 W m^{-2} Hz^{-1}<br>607.71 W m^{-3} Hz^{-1}
Wu et al *Energy Environ. Sci.* (2023)[13]	Charge migration	PET/PU foam	---	1.5 Hz	78.78 cm^2<br>~87.05 cm^2	14.1 W m^{-2}<br>9.4 W m^{-2} Hz^{-1}<br>~850.68 W m^{-3} Hz^{-1}
Li et al *Energy Environ. Sci.* (2023)[14]	Electrostatic breakdown	PTFE/PU foam	1.023	0.5 Hz	---	5.99 W m^{-2}<br>(total area)<br>11.98 W m^{-2} Hz^{-1}
This work	Ternary dielectric triboelectrification effect	PTFE/ PF/Pu foam	1.00375	2 Hz	33.175 cm^2<br>33.491 cm^3	32.121 W m^{-2}<br>16.061 W m^{-2} Hz^{-1}<br>1591 W m^{-3} Hz^{-1}

Table S2. The detailed motion parameters of sliding FEM-TENG.

Test parameters	Amplitude	Acceleration	Deceleration	Terminal waiting time	Average traction
**Numerical size**	**10 cm**	**0.5 m s^{-2}**	**0.5 m s^{-2}**	**50 μs**	**6.06 N**

Table S3. Voltage crest factor of sliding FEM-TENG at different sliding speeds.

Sliding speed ($m\ s^{-1}$)	Peak voltage (V)	Root mean square of steady voltage (V)	Crest factor
**0.05**	**4614.99**	**4094.72**	**1.1271**
**0.10**	**3842.41**	**3558.75**	**1.0797**
**0.15**	**3631.11**	**3400.57**	**1.0678**
**0.20**	**3278.03**	**3138.08**	**1.0446**

Table S4. Voltage crest factor of rotating FEM-TENG at different rotating speeds.

Rotating speed (rpm)	Peak voltage (V)	Root mean square of steady voltage (V)	Crest factor
30	2928.34	2838.11	1.0318
60	3147.54	3075.67	1.0234
90	2897.46	2850.23	1.0166
120	2899.99	2872.06	1.0097

Table S5. Current crest factor of rotating FEM-TENG at different rotating speeds.

Rotating speed (rpm)	Peak current (μA)	Root mean square of steady current (μA)	Crest factor
30	17.67	15.02	1.176
60	31.85	30.06	1.060
90	35.21	34.91	1.0086
120	40.10	39.95	1.00375

Table S6. Comparison of charge density per round with the latest and most typical rotating DC-TENGs [1,3,5,7,10-16].

Article (rotation mode)	Type	Number of integrated units	Volume and area of triboelectric layer	Charge output	Charge density per round
Liu et al *Sci. Adv.* (2019)[15]	Electrostatic breakdown	60	314 cm^2 189.97 cm^3	1.781 μC/r	0.057 mC m^{-2} 9.375 mC m^{-3}
Li et al *Energy Environ. Sci.* (2022)[3]	Phase control	6	706.5 cm^2 706.5 cm^3	14 μC/r	0.198 mC m^{-2} 19.8 mC m^{-3}
Zhou et al *Adv. Energy Mater.* (2020)[1]	Electrostatic breakdown	18	153.86 cm^2 152.74 cm^3	3.5 μC/r	0.227 mC m^{-2} 22.9 mC m^{-3}
Chen et al *Energy Environ. Sci.* (2021)[7]	Phase control	9	113 cm^2 170.8 cm^3	4.252 μC/r	0.376 mC m^{-2} 24.9 mC m^{-3}
Wu et al *Energy Environ. Sci.* (2023)[13]	Charge migration	3	78.78 cm^2 ~87.05 cm^3	3.516 μC/r	0.446 mC m^{-2} 40.4 mC m^{-3}
Zeng et al *Adv. Mater.* (2023)[5]	Electrostatic breakdown	1	157 cm^2 64.527 cm^3	9 μC/r	0.573 mC m^{-2} 139.5 mC m^{-3}
Du et al *Adv. Funct. Mater.* (2022)[10]	Mechanical time-delay switch	8	96 cm^2 ~96 cm^3	25 μC/r	2.604 mC m^{-2} 260.4 mC m^{-3}
Zhao et al *Nat. Commun.* (2020)[16]	Electrostatic breakdown	50	25.133 cm^2 26.39 cm^3	6.67 μC/r	2.65 mC m^{-2} 252.7 mC m^{-3}
Li et al *Adv. Energy Mater.* (2023)[11]	Ternary dielectric triboelectrification effect	32	25.655 cm^2 25.835 cm^3	10.01 μC/r	3.902 mC m^{-2} 387.5 mC m^{-3}
Li et al *Energy Environ. Sci.* (2023)[14]	Electrostatic breakdown	36	---	---	6.15 mC m^{-2} (total area)
Li et al *Energy Environ. Sci.* (2023)[12]	Ternary dielectric triboelectrification effect	32	26.267 cm^2 26.582 cm^3	18.7 μC/r	7.12 mC m^{-2} 703.5 mC m^{-3}
This work	Ternary dielectric triboelectrification effect	8	33.175 cm^2 33.491 cm^3	32.54 μC/r	9.807 mC m^{-2} 971.6 mC m^{-3}

Table S7. Comparison of energy conversion efficiency with the most representative rotary TENG [12,17-20].

Article (rotary TENG)	Type (AC/DC)	Rotation speed	Energy conversion efficiency η
**Cho et al** ***Nano Energy*** **(2022)[17]**	**Hybrid generator based on TENG and EMG (AC)**	**500 rpm**	**8.75%**
**Li et al** ***Appl. Energy*** **(2022)[18]**	**Breeze-driven TENG (AC)**	**4 m s^{-1}**	**12.06%**
**Han et al** ***Adv. Funct. Mater.*** **(2022)[19]**	**Rabbit fur-based soft-contact TENG (AC)**	**6 m s^{-1}**	**15.4%**
**He et al** ***Research*** **(2022)[20]**	**Liquid lubrication promoted TENG (AC)**	**60 rpm**	**48.61%**
**Li et al** ***Energy Environ. Sci.*** **(2023)[12]**	**Ternary DC-TENG (DC)**	**120 rpm**	**22.84%**
**This work**	**Field emission model-based DC-TENG (DC)**	**120 rpm**	**14.27%**

Table S8. Voltage crest factor of FEM-TENG before and after the stability test.

Situation	Peak voltage (V)	Root mean square of steady voltage (V)	Crest factor
**Before stability test**	**3122.50**	**3088.37**	**1.0111**
**After stability test**	**3129.20**	**3095.59**	**1.0109**

Table S9. Wind speed under different input voltages.

Input voltage of blower (V)	Wind speed range (m s^{-1})	Average wind speed (m s^{-1})
150	1.2~2.4	1.8
160	1.8~3	2.4
170	2.4~3.6	3.0
180	3~4.2	3.6

Table S10. Current crest factor of FEM-TENG under different wind speeds.

Average wind speed (m s^{-1})	Peak current (μA)	Root mean square of steady current (μA)	Crest factor
**1.8**	**10.65**	**10.02**	**1.063**
**2.4**	**12.53**	**12.14**	**1.032**
**3.0**	**16.35**	**15.73**	**1.039**
**3.6**	**20.2**	**19.55**	**1.033**

Table S11. Voltage crest factor of FEM-TENG under different wind speeds.

Average wind speed (m s^{-1})	Peak voltage (V)	Root mean square of steady voltage (V)	Crest factor
1.8	2763.87	2624.43	1.053
2.4	2782.57	2714.53	1.025
3.0	2772.01	2694.81	1.029
3.6	2837.30	2769.62	1.024

Table S12. Power density comparison with state-of-the-art rotary wind-driven TENGs [21-28].

Article	AC/DC	Working range (Optimum speed)	Wind or water powered	Area of total triboelectric layer (cm^2)	Power density
**Pang et al** ***Adv. Funct. Mater.*** **(2021)[21]**	**AC**	**0.8~1.4 Hz (1.1 Hz)**	**Water**	**218.5**	**0.0339 W m^{-2} (Average)**
**Yong et al** ***Adv. Energy Mater.*** **(2022)[22]**	**AC**	**2~16.2 m s^{-1} (7.9 m s^{-1})**	**Wind**	**112.59**	**0.444 W m^{-2} (Peak)**
**You et al** ***NANO ENERGY*** **(2023)[23]**	**DC**	**4~7 m s^{-1} (7 m s^{-1})**	**Wind**	**2.5**	**0.78 mW m^{-2} (Average)**
**Han et al** ***Adv. Funct. Mater.*** **(2022)[24]**	**AC**	**1~6 m s^{-1} (6 m s^{-1})**	**Wind**	**51.7**	**2.3 W m^{-2} (Peak)**
**Zhang et al** ***ACS Energy Letters*** **(2021)[25]**	**AC**	**20~100 m s^{-1} (20 m s^{-1})**	**Wind**	**32**	**2.96 W m^{-2} (Average)**
**He et al** ***Adv. Mater.*** **(2023)[26]**	**AC**	**1~9 m s^{-1} (8 m s^{-1})**	**Wind**	**---**	**4.4 W m^{-2} (Average)**
**Mu et al** ***Adv. Funct. Mater.*** **(2023)[27]**	**AC**	**3.5~5.5 m s^{-1} (3.5 m s^{-1})**	**Wind**	**171**	**4.713 W m^{-2} (Peak)**
**Long et al** ***Nat. Commun.*** **(2021)[28]**	**AC**	**3~7 m s^{-1} (7 m s^{-1})**	**Wind**	**27.98**	**5.967 W m^{-2} (Peak)**
**This work**	**DC**	**1.2~4.2 m s^{-1} (3.6 m s^{-1})**	**Wind**	**33.175**	**8.461 W m^{-2} (Average)**

Table S13. Charging rates of various capacitors when FEM-TENG is driven by wind and stepper motors respectively.

Capacitors	Times	The equivalent charging rate	Ways to drive TENG
220 μF	0.616 s	1785.71 μC s^{-1}	Stepper motor
	1.45 s	758.62 μC s^{-1}	Wind
470 μF	1.64 s	1432.93 μC s^{-1}	Stepper motor
	7.37 s	318.86 μC s^{-1}	Wind
1000 μF	2.73 s	1831.50 μC s^{-1}	Stepper motor
	10.45 s	478.47 μC s^{-1}	Wind
2200 μF	5.36 s	2052.24 μC s^{-1}	Stepper motor
	11.58 s	949.91 μC s^{-1}	Wind
3300 μF	7.66 s	2154.05 μC s^{-1}	Stepper motor
	19.52 s	845.29 μC s^{-1}	Wind
4700 μF	10 s	2350.00 μC s^{-1}	Stepper motor
	25 s	940.00 μC s^{-1}	Wind

Note S1: Working mechanism of the sliding FEM-TENG before reaching charge saturation state.

As shown in Fig. S5A, when the slider slides to the right, PTFE and PU foam rub against the polyester fur respectively. At this point, based on the ternary dielectric triboelectrification effect, given the charge affinities of the three materials as PTFE>polyester fur>PU foam, PTFE gains electrons while PU foam loses electrons. The slider moves forward and away from its initial position, creating electrostatic induction on the slider at this moment (Figure S5B). As a consequence of the negative charges induced by the back electrodes of PA and the positive charges induced by the back electrodes of PTFE, a constant current is generated in the external circuit. The PTFE is then slid over the polyester fur that had rubbed against the PU foam, as shown in Figure S5C. Since the electronegativity of polyester fur is weaker than that of PTFE, the electrons on the polyester fur are "transferred" to PTFE. In general, the three materials once again produce triboelectric effects at this time. As the slider continues to slide, in order to balance the potential difference between PTFE and PU foam, electrons flow from the back electrodes of PTFE to the back electrodes of PU foam, continuously forming a DC output on the external circuit (Figure S5D). It is worth noting that we put quotation marks around the word "transfer". In fact, charge generally does not transfer on insulating materials. The "charge transfer" here is due to the friction between the three materials, which causes friction charging due to the difference in electronegativity.

Note S2: The calculation of friction coefficient.

The friction coefficient (μ) of the sliding FEM-TENG is calculated as follows:

$$\mu = \frac{F_{Average}}{F_{Supporting\ force}} \tag{1}$$

Where $F_{Average}$ is the average value of the traction force measured by the digital force gauge connected to the slider (6.06433 N). In **Fig. S12**, the slider is pulled by the linear motor and slides horizontally on the stator at a constant speed. The digital force gauge continuously measures the traction force during operation. $F_{Supporting\ force}$ represents the support force exerted by the stator on the slider, which is approximately equal to the pressure of the weight on the slider (19.6 N). Therefore, the friction coefficient between the slider and stator (polyester fur) of the 2-unit FEM-TENG is 0.3094.

Note S3: Energy conversion efficiency of FEM-TENG.

According to the principle of triboelectric charging, the energy generated by the device during one rotation is mainly divided into three types: electrical energy output E_o, rotational kinetic energy E_k, and energy used to resist friction E_f [12,20]. Therefore, the energy conversion efficiency η can be described as follows:

$$\eta = \frac{E_o}{E_o+E_k+E_f} \tag{2}$$

$$E_o = P_A \cdot T \tag{3}$$

$$E_k = \frac{1}{2} I\omega^2 = \frac{1}{4} m(R_1^2 + R_2^2)\omega^2 \tag{4}$$

Where P_A is the average power generated by FEM-TENG, which is approximately 0.1066 W. And T is the time for one rotation, which is 0.5 s. I is the rotational inertia, m is the weight of the device (140 g) and the ω is the angular velocity (4π). R_1 and R_2 are the inner and outer radius of the rotor (R_1 is 10 mm, and R_2 is 100 mm). ω is the angular velocity, which is 4π.

As for the energy used to resist friction E_f, it is more convenient to calculate it using friction moment.

$$M = \int \boldsymbol{r} \times d\boldsymbol{f} = \int r df = \int r\mu dF_N = \frac{2}{3}\mu F_N \frac{R_2^3-R_1^3}{R_2^2-R_1^2} \tag{5}$$

$$E_f = \int_0^{2\pi} M d\theta = \frac{4}{3}\pi\mu F_N \frac{R_2^3-R_1^3}{R_2^2-R_1^2} \tag{6}$$

Where the radius $\boldsymbol{r}$ is perpendicular to the friction force $\boldsymbol{f}$. μ is the dynamic friction coefficient (0.3094) and the F_N is the positive pressure (20 N). Therefore, the energy conversion efficiency η is calculated as follows.

$$\eta = \frac{E_o}{E_o+E_k+E_f} = \frac{P_A \cdot T}{P_A \cdot T + \frac{1}{4}m(R_1^2+R_2^2)\omega^2 + \frac{4}{3}\pi\mu F_N \frac{R_2^3-R_1^3}{R_2^2-R_1^2}} \tag{7}$$

Therefore, the energy conversion efficiency of FEM-TENG is 14.27%.

Note S4: Safety risks posed by needle-embedded back electrodes.

The safety risks of needle-embedded back electrodes from the following three aspects:

1. **Risks of embedded back electrodes to devices.** Although embedding steel needles into thick dielectric films can increase the leakage current of dielectric materials, it requires high skills in the production process. If the needles are inserted too deeply, the higher voltage in the FEM-TENG will cause dielectric breakdown of the dielectric materials, causing permanent damage to the materials and the device. As a result, the output of the device will be seriously affected. Therefore, to avoid the safety risks of the embedded back electrodes to the device, we recommend that the experimenter should use a high-voltage power supply to provide voltage to the dielectric film with the embedded back electrodes before testing the output. The voltage provided should be gradually increased to ensure that the device can withstand a high voltage of more than 3000 V.
2. **Risks of embedded back electrodes to experimenters.** The use of steel needles also increases the risk of electric shock to experimenters. During the experiment, a large amount of electric charge accumulates at the tip of the steel needle, which will cause strong air breakdown between the needle and the exposed skin of the experimenter who actively approaches. Therefore, during the test, the experimenter must wear insulating gloves and short-circuit the output end of the device before picking it up. In addition, during the device manufacturing process, the experimenter should be careful not to be stabbed by the steel needle.
3. **Risks of embedded back electrodes to experimental equipment.** The high-voltage output of FEM-TENG and the design of buried needle electrodes place high demands on testing instruments. The experimental equipment should first be grounded to prevent high voltage discharge during the experiment. Moreover, the range of the test equipment should be as large as possible to prevent the device output from exceeding the range and causing a short circuit. For test equipment with a smaller range, it is more appropriate to consider using the voltage division method to measure voltage.

References

[1] L. Zhou, D. Liu, S. Li et al., "Rationally designed dual-mode triboelectric nanogenerator for harvesting mechanical energy by both electrostatic induction and dielectric breakdown effects," *Advanced Energy Materials*, vol. 10, pp. 2000965, 2020.

[2] Z. Wu, S. Wang, Z. Cao et al., "Rotary disk multi-phase freestanding-electret generator with enhanced power and low ripple output," *Nano Energy*, vol. 83, pp. 105787, 2021.

[3] X. Li, C. Zhang, Y. Gao et al., "A highly efficient constant-voltage triboelectric nanogenerator," *Energy & Environmental Science*, vol. 15, pp. 1334-1345, 2022.

[4] H. Ryu, J. Lee, U. Khan et al., "Sustainable direct current powering a triboelectric nanogenerator via a novel asymmetrical design," *Energy & Environmental Science*, vol. 11, pp. 2057-2063, 2018.

[5] Q. Zeng, A. Chen, X. Zhang et al., "A dual-functional triboelectric nanogenerator based on the comprehensive integration and synergetic utilization of triboelectrification, electrostatic induction, and electrostatic discharge to achieve alternating current/direct current convertible outputs," *Advanced Materials*, vol. 35, pp. 2208139, 2023.

[6] H. Wu, S. Wang, Z. Wang, & Y. Zi, "Achieving ultrahigh instantaneous power density of 10 MW/m-2 by leveraging the opposite-charge-enhanced transistor-like triboelectric nanogenerator (OCT-TENG)," *Nature Communications*, vol. 12, pp. 5470, 2021.

[7] P. Chen, J. An, R. Cheng et al., "Rationally segmented triboelectric nanogenerator with a constant direct-current output and low crest factor," *Energy & Environmental Science*, vol. 14, pp. 4523-4532, 2021.

[8] Z. Wang, Z. Zhang, Y. Chen et al., "Achieving an ultrahigh direct-current voltage of 130 V by semiconductor heterojunction power generation based on the tribovoltaic effect," *Energy & Environmental Science*, vol. 15, pp. 2366-2373, 2022.

[9] Z. Zhang, Z. Wang, Y. Chen et al., "Semiconductor contact-electrification-dominated tribovoltaic effect for ultrahigh power generation," *Advanced Materials*, vol. 34, pp. 2200146, 2022.

[10] Y. Du, S. Fu, C. Shan et al., "A novel design based on mechanical time-delay switch and charge space accumulation for high output performance direct-current triboelectric nanogenerator," *Advanced Functional Materials*, vol. 32, pp. 2208783, 2022.

[11] Q. Li, Y. Hu, Q. Yang et al., "A robust constant-voltage DC triboelectric nanogenerator using the ternary dielectric triboelectrification effect," *Advanced Energy Materials*, vol. 13, pp. 2202921, 2023.

[12] Q. Li, S. Fu, X. Li et al., "Overall performance improvement of direct-current triboelectric nanogenerators by charge leakage and ternary dielectric evaluation," *Energy & Environmental Science*, vol. 16, pp. 3514-3525, 2023.

[13] H. Wu, J. Wang, S. Fu et al., "A constant current triboelectric nanogenerator

achieved by hysteretic and ordered charge migration in dielectric polymers," *Energy & Environmental Science*, vol. 16, pp. 5144-5153, 2023.
[14] K. Li, C. Shan, S. Fu et al., "High efficiency triboelectric charge capture for high output direct current electricity," *Energy & Environmental Science*, vol. 17, pp. 580-590, 2024.
[15] D. Liu, X. Yin, H. Guo et al., "A constant current triboelectric nanogenerator arising from electrostatic breakdown," *Science Advances*, vol. 5, pp. eaav6437, 2019.
[16] Z. Zhao, Y. Dai, D. Liu et al., "Rationally patterned electrode of direct-current triboelectric nanogenerators for ultrahigh effective surface charge density," *Nature Communications*, vol. 11, pp. 6186, 2020.
[17] H. Cho, I. Kim, J. Park and D. Kim, "A waterwheel hybrid generator with disk triboelectric nanogenerator and electromagnetic generator as a power source for an electrocoagulation system," *Nano Energy*, vol. 95, pp. 107048, 2022.
[18] X. Li, Y. Cao, X. Yu et al., "Breeze-driven triboelectric nanogenerator for wind energy harvesting and application in smart agriculture," *Applied Energy*, vol. 306, pp. 117977, 2022.
[19] J. Han, Y. Feng, P. Chen et al., "Wind-driven soft-contact rotary triboelectric nanogenerator based on rabbit fur with high performance and durability for smart farming," *Advanced Functional Materials*, vol. 32, pp. 2108580, 2022.
[20] W. He, W. Liu, S. Fu et al., "Ultrahigh performance triboelectric nanogenerator enabled by charge transmission in interfacial lubrication and potential decentralization design", Research, vol. 2022, pp. 11, 2022. Article ID 9812865.
[21] H. Pang, Y. Feng, J. An et al., "Segmented swing-structured fur-based triboelectric nanogenerator for harvesting blue energy toward marine environmental applications," *Advanced Functional Materials*, vol. 31, pp. 2106398, 2021.
[22] S. Yong, H. Wang, Z. Lin et al., "Environmental self-adaptive wind energy harvesting technology for self-powered system by triboelectric-electromagnetic hybridized nanogenerator with dual-channel power management topology," *Advanced Energy Materials*, vol. 12, pp. 2202469, 2022.
[23] Z. You, X. Wang, F. Lu et al., "An organic semiconductor/metal Schottky heterojunction based direct current triboelectric nanogenerator windmill for wind energy harvesting," *Nano Energy*, vol. 109, pp. 108302, 2023.
[24] J. Han, Y. Feng, P. Chen et al., "Wind-driven soft-contact rotary triboelectric nanogenerator based on rabbit fur with high performance and durability for smart farming," *Advanced Functional Materials*, vol. 32, pp. 2108580, 2022.
[25] C. Zhang, Y. Liu, B. Zhang et al., "Harvesting wind energy by a triboelectric nanogenerator for an intelligent high-speed train system," *Acs Energy Letters*, vol. 6, pp. 1490-1499, 2021.
[26] W. He, C. Shan, S. Fu et al., "Large harvested energy by self-excited liquid suspension triboelectric nanogenerator with optimized charge transportation behavior," *Advanced Materials*, vol. 35, pp. 2209657, 2023.
[27] Q. Mu, W. He, C. Shan et al., "Achieving high-efficiency wind energy harvesting triboelectric nanogenerator by coupling soft contact, charge space accumulation,

and charge dissipation design," *Advanced Functional Materials*, vol. 34, pp. 2309421, 2023.

[28] L. Long, W. Liu, Z. Wang et al., "High performance floating self-excited sliding triboelectric nanogenerator for micro mechanical energy harvesting," *Nature Communications*, vol. 12, pp. 4689, 2021.
